# Mitochondrial sulfide promotes life span and health span through distinct mechanisms in developing versus adult treated *Caenorhabditis elegans*

**DOI:** 10.1073/pnas.2216141120

**Published:** 2023-07-31

**Authors:** Adriana Raluca Vintila, Luke Slade, Michael Cooke, Craig R. G. Willis, Roberta Torregrossa, Mizanur Rahman, Taslim Anupom, Siva A. Vanapalli, Christopher J. Gaffney, Nima Gharahdaghi, Csaba Szabo, Nathaniel J. Szewczyk, Matthew Whiteman, Timothy Etheridge

**Affiliations:** ^a^Public Health and Sport Sciences, Faculty of Health and Life Sciences, University of Exeter, Exeter EX1 2LU, United Kingdom; ^b^University of Exeter Medical School, Faculty of Health and Life Sciences, University of Exeter, Exeter EX1 2LU, United Kingdom; ^c^Medical Research Council Versus Arthritis Centre for Musculoskeletal Ageing Research, Nottingham Biomedical Research Center, School of Medicine, Royal Derby Hospital, University of Nottingham, Derby DE22 3DT, United Kingdom; ^d^School of Chemistry and Biosciences, Faculty of Life Sciences, University of Bradford, Bradford BD7 1DP, United Kingdom; ^e^Department of Chemical Engineering, Texas Tech University, Lubbock, TX 79409; ^f^Department of Electrical Engineering, Texas Tech University, Lubbock, TX 74909; ^g^Lancaster University Medical School, Lancaster University, Lancaster LA1 4YW, United Kingdom; ^h^Chair of Pharmacology, Section of Medicine, University of Fribourg, Fribourg CH-1700, Switzerland; ^i^Ohio Musculoskeletal and Neurologic Institute, Heritage College of Osteopathic Medicine, Ohio University, Athens, OH 45701

**Keywords:** health span, longevity, mitochondria, transcriptomics, H_2_S

## Abstract

Living longer without simultaneously extending years spent in good health (“health span”) is an increasing societal burden, demanding new therapeutic strategies. Hydrogen sulfide (H_2_S) can correct disease-related mitochondrial metabolic deficiencies, and supraphysiological H_2_S concentrations can pro health span. However, the efficacy and mechanisms of mitochondrion-targeted sulfide delivery molecules (mtH_2_S) administered across the adult life course are unknown. Using a *Caenorhabditis elegans* aging model, we compared untargeted H_2_S (NaGYY4137, 100 µM and 100 nM) and mtH_2_S (AP39, 100 nM) donor effects on life span, neuromuscular health span, and mitochondrial integrity. H_2_S donors were administered from birth or in young/middle-aged animals (day 0, 2, or 4 postadulthood). RNAi pharmacogenetic interventions and transcriptomics/network analysis explored molecular events governing mtH_2_S donor-mediated health span. Developmentally administered mtH_2_S (100 nM) improved life/health span vs. equivalent untargeted H_2_S doses. mtH_2_S preserved aging mitochondrial structure, content (citrate synthase activity) and neuromuscular strength. Knockdown of H_2_S metabolism enzymes and FoxO/*daf-16* prevented the positive health span effects of mtH_2_S, whereas DCAF11/*wdr-23* – Nrf2/*skn-1* oxidative stress protection pathways were dispensable. Health span, but not life span, increased with all adult-onset mtH_2_S treatments. Adult mtH_2_S treatment also rejuvenated aging transcriptomes by minimizing expression declines of mitochondria and cytoskeletal components, and peroxisome metabolism hub components, under mechanistic control by the *elt-6*/*elt-3* transcription factor circuit. H_2_S health span extension likely acts at the mitochondrial level, the mechanisms of which dissociate from life span across adult vs. developmental treatment timings. The small mtH_2_S doses required for health span extension, combined with efficacy in adult animals, suggest mtH_2_S is a potential healthy aging therapeutic.

## Significance

Deteriorating health across the life course is a major societal burden, and effective therapeutics are lacking. Mitochondrial decline has long been associated with age-related health loss. We show that small, clinically meaningful doses of a mitochondrion-targeting sulfur donor (AP39) extend *Caenorhabditis elegans* health in older age, which act by maintaining mitochondrial integrity. Adult onset of AP39 delivery, when mitochondrial and cell structural dysfunction are already manifested, also promoted healthy aging. Distinct association of health span extension with mitochondria, cytoskeletal and peroxisome molecular profiles, under regulation of the *elt-6*/*elt-3* transcription factor regulatory circuit, further distinguished adult-onset AP39 therapy. Our results establish a framework for forward translating mitochondrial sulfide as a potentially viable healthy aging intervention in mammals.

Medical advances mean humans are living longer but are also spending longer in a frail “poor health” state (1, 2), with large burdens on healthcare systems and quality of life (3). Since most age-related healthcare costs and patient frailty occur in the later years of life (1, 2), interventions that increase life span without simultaneously increasing health span would be detrimental to the aging process. Studies often report life span–extending therapeutics in lower organisms (4, 5), but a significant caveat is the general assumption that increasing longevity also prolongs the duration spent in a healthy state (termed “health span”). While it is largely unknown whether most conditions that extend life span also increase health span, evidence indicates dissociation between the two (6). For example, all long-lived *Caenorhabditis elegans* mutants examined to date spend a longer time in an aged frail condition (7); the same phenomenon reported in long-living humans (1, 2). Therapeutic approaches that extend healthy years, rather than life span alone, thus hold considerable socioeconomic potential.

Hydrogen sulfide (H_2_S) was one of the essential ingredients required for life to emerge on Earth (8, 9) and has emerged as an important, physiologically relevant signaling molecule. When applied exogenously, H_2_S treatments, usually in the form of crude impure sulfide salts at supraphysiological concentrations (e.g. >100 µM), confer cytoprotective properties across various pathophysiological states (9–14), including age-associated diseases (15, 16). Accordingly, 100 to 150 µM concentrations of untargeted H_2_S donors such as GYY4137 and FW1256 extend both life span (17–19) and health span (20) in *C. elegans* when administered from birth. However, several essential biochemical processes are established during development that program subsequent adult behavior. For example, developmental starvation cements locomotion circuitry that impacts adult foraging behavior (21), and developmentally established mitochondrial dynamics determine rates of adult respiration and aging (22). Additionally, life-extending mitochondrial interventions in *C. elegans* currently require administration on or before the developmental larval stages (23). Metabolic patterns set during developmental H_2_S treatments might, therefore, mediate life span and health span extension. The efficacy of H_2_S administered during “normal” stochastic aging thus warrants investigation to understand the viability of adult H_2_S-based therapies.

Several cellular processes are responsive to H_2_S that can regulate H_2_S-mediated longevity (18), yet the mechanisms governing H_2_S-mediated health span are undefined. Increasing evidence supports a mitochondrion-centric mode of H_2_S action across cell types and pathologies. Current dogma suggests H_2_S donates electrons to the mitochondrial electron transport chain, inhibits mitochondrial cAMP phosphodiesterases, facilitates mitochondrial DNA repair, promotes mitochondrial antioxidant protection, and augments mitochondrial respiration/ATP production (reviewed in ref. 24). Moreover, mitochondrial loss is one of the nine hallmarks of aging (25) and is the earliest detectable subcellular structural change during *C. elegans* aging (26), preceding physiological decline (27). As such, therapies that exploit positive H_2_S effects on mitochondria represent an attractive antiaging strategy. The mitochondrial sulfide delivery molecule (mtH_2_S), AP39, exploits mitochondrial membrane potential by utilizing a TPP^+^ motif to localize H_2_S to the mitochondria and protect against cellular injury [e.g., glucose oxidase–induced mitochondrial dysfunction (28)] vs. equal doses of untargeted H_2_S donors. Consequently, unlike untargeted H_2_S compounds (e.g., GYY4137, FW1256) with supraphysiological effective doses (17–20), mtH_2_S displays potency at concentrations several orders of magnitude lower in *C. elegans* disease models (13, 14). Whether such phenomena occur in the aging context is unknown; however, mtH_2_S is plausibly responsible for longevity and health span extension reported following larger untargeted H_2_S doses.

This study, therefore, investigated the efficacy of a mtH_2_S (AP39) for promoting health span via mitochondrion-mediated effects vs. untargeted H_2_S donors, using *C. elegans* as an aging model. Given the unknown capacity of H_2_S as an efficacious therapy in aging adults, we also examined health span effects of adult-onset H_2_S treatments. Using functional pharmacogenetic approaches, we provide evidence that mtH_2_S is a requirement for, and site of action of, H_2_S-mediated health span promotion. Importantly, mtH_2_S increases health span when administered to young and middle-aged adults, and this adult treatment effect is clearly reflected at the transcriptomic level compared to developmental mtH_2_S administration, under the control of a GATA family of transcription factors. These findings strongly suggest that augmentation of mitochondrial sulfide may represent a druggable target and translatable therapeutic approach to maintaining health with advancing age, at time points where the negative effects of aging already manifest.

## Results

### mtH_2_S Increases *C. elegans* Life span and Neuromuscular Health span.

**Fig. 1.**
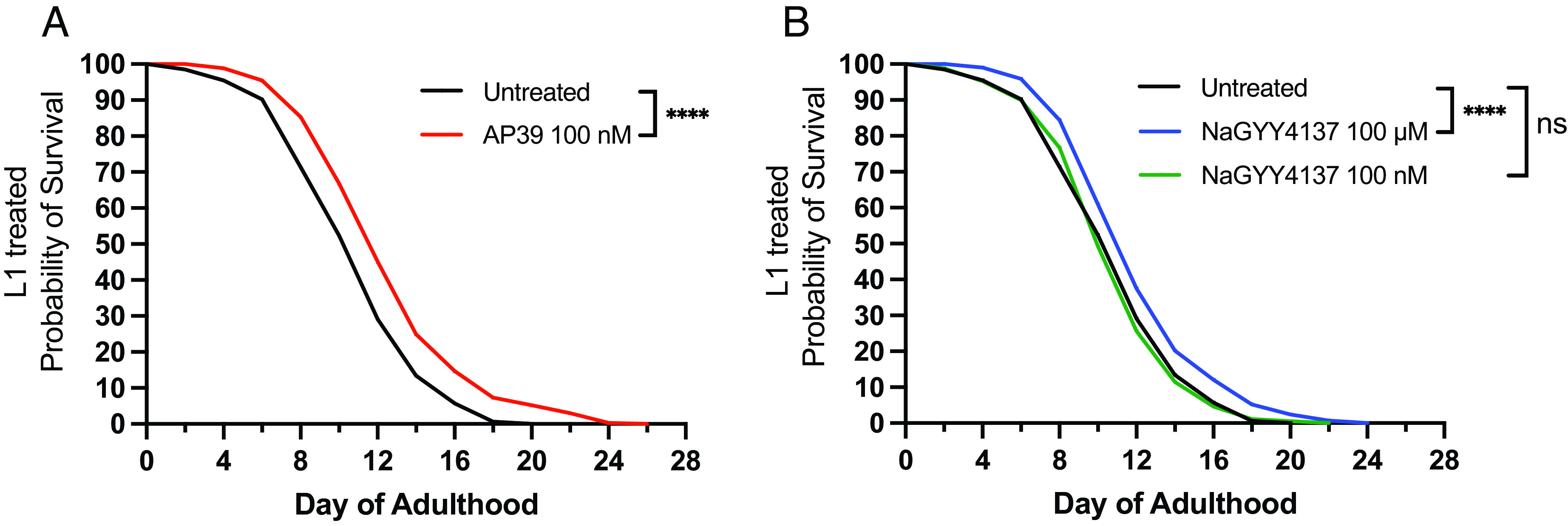
Lower doses of mitochondrion-targeted H_2_S extend life span. (*A*) *C. elegans* life span is significantly increased with higher (100 µM), but not lower (100 nM) treatment with the untargeted H_2_S donor, NaGYY4137 when administered from L1 larval stage across the entire lifecourse. (*B*) Conversely, lower doses (100 nM) of mitochondrion-targeted H_2_S (AP39) extend life span. Life span curves represent the average of three biological replicates (total ~300 to 600 animals per condition). **** denotes significant difference vs. untreated (0.01% DMSO) wild-type controls (*P* < 0.0001). ns, nonsignificant.

We first investigated the effects of mitochondrion-targeted and nontargeted H_2_S donors on *C. elegans* life span. Dosing L1 larvae with the untargeted sulfide donor NaGYY4137 (100 µM) increased maximal life span by 20 % (*P* < 0.0001) (Fig. 1*A*), which is comparable to previous studies using GYY4137 (morpholine salt) and related compounds (18, 19). In sharp contrast, the mitochondrion-targeted sulfide delivery molecule (mtH_2_S) AP39 significantly increased life span at 1000-fold lower doses (100 nM) by 30% (*P* < 0.0001) (Fig. 1*B*), whereas equivalent 100 nM doses of untargeted NaGYY4137 had no significant effect on *C. elegans* life span extension (Fig. 1*A*).

**Fig. 2.**
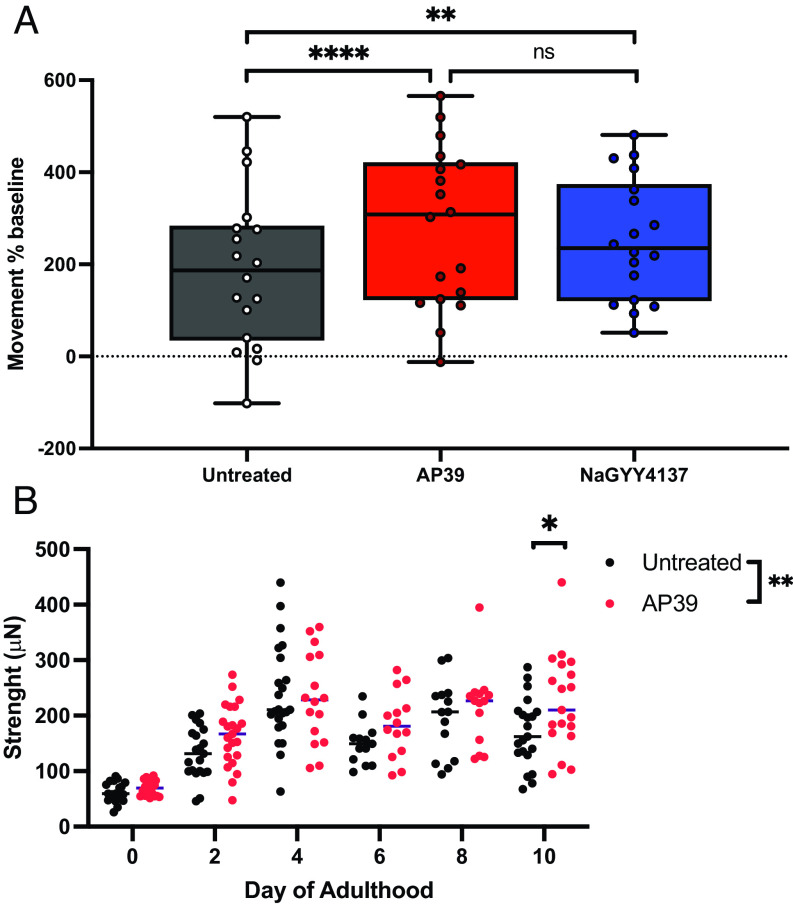
Mitochondrion-targeted H_2_S extends movement rate and maximal strength indices of health span. (*A*) Animal movement rate is increased across the entire lifecourse with both lower dose (100 nM) mitochondrion-targeted H_2_S (AP39) and higher dose (100 µM) untargeted H_2_S (NaGYY4137) when administered from L1 larval stage until death. Movement rates as a % change from day 0 baselines, across days 0, 2, 4, 8, 12, and 16 postadulthood, are presented as area under the curve. (*B*) Lower dose (100 nM) mitochondrion-targeted H_2_S maintains *C. elegans* maximal strength producing ability in later life (day 10 postadulthood), measured using our microfluidic NemaFlex device. Data presented are mean ± SD, n = 90 per condition, across 3 biological replicates. *P < 0.05, **P < 0.01 and ****P < 0.0001 denotes significant difference vs. untreated (0.01% DMSO) wild-type controls.

We next assessed movement rates on days 0, 2, 4, 8, 12, and 16 of life span as a robust proxy of overall animal health (29–31) and, therefore, health span. Using wMicroTracker to measure prolonged movement capacity beyond standard thrash assays (32), wild-type movement capacity peaked at day 4 of adulthood (+97% vs. day 0 baseline), as previously published (33, 34), and progressively declined thereafter to a nadir of −26% at day 16 (*SI Appendix*, Fig. S1). At greatly different doses, 100 µM NaGYY4137 H_2_S and 100 nM mtH_2_S increased total animal movement rates across the life course (*P* < 0.001), using area under the curve analysis of movement across the life course, as previously published (30) (Fig. 2*A*). Post hoc analysis showed significant health span extension in mtH_2_S (100 nM)-treated animals up to day 16 postadulthood compared to day 12 postadulthood during NaGYY4137 treatments (*SI Appendix*, Fig. S1). Loss of neuromuscular strength is also one of the strongest correlates of all-cause mortality in humans (29, 35), leading us to employ our “NemaFlex” device (36–38) to examine neuromuscular strength changes across age. As with movement rates (*SI Appendix*, Fig. S1), wild-type strength capacity increased between days 0 and 4 adulthood and declined thereafter. Conversely, treatment with mtH_2_S (100 nM) improved strength production across days 0–10 postadulthood (*P* < 0.001), with a significant 20% strength increase vs. wild-type at day 10 (Fig. 2*B*). Additionally, while the observed effect sizes of mtH_2_S are comparable to those reported for other life span–extending compounds (39–41), the improvements we observed are modest. We, therefore, directly compared mtH_2_S to a recently published life span and health span improving drug, rilmenidine (42), using our microfluidic “Nemalife” health span device, and found both compounds extended life span and health span to similar degrees using our microfluidic approach (*SI Appendix*, Fig. S2). Rilmendine also has no effect on neuromuscular health parameters in early life (42) but rather manifest in older age time points, as observed herein for mtH_2_S. Collectively, these data strongly suggest that H_2_S effects on health span are likely mediated through mitochondrial effects which, although modest, may be highly beneficial, since aging is also associated with a later life loss of prolonged movement and strength-producing capacity.

### mtH_2_S Maintains Mitochondrial Structure and Content.

**Fig. 3.**
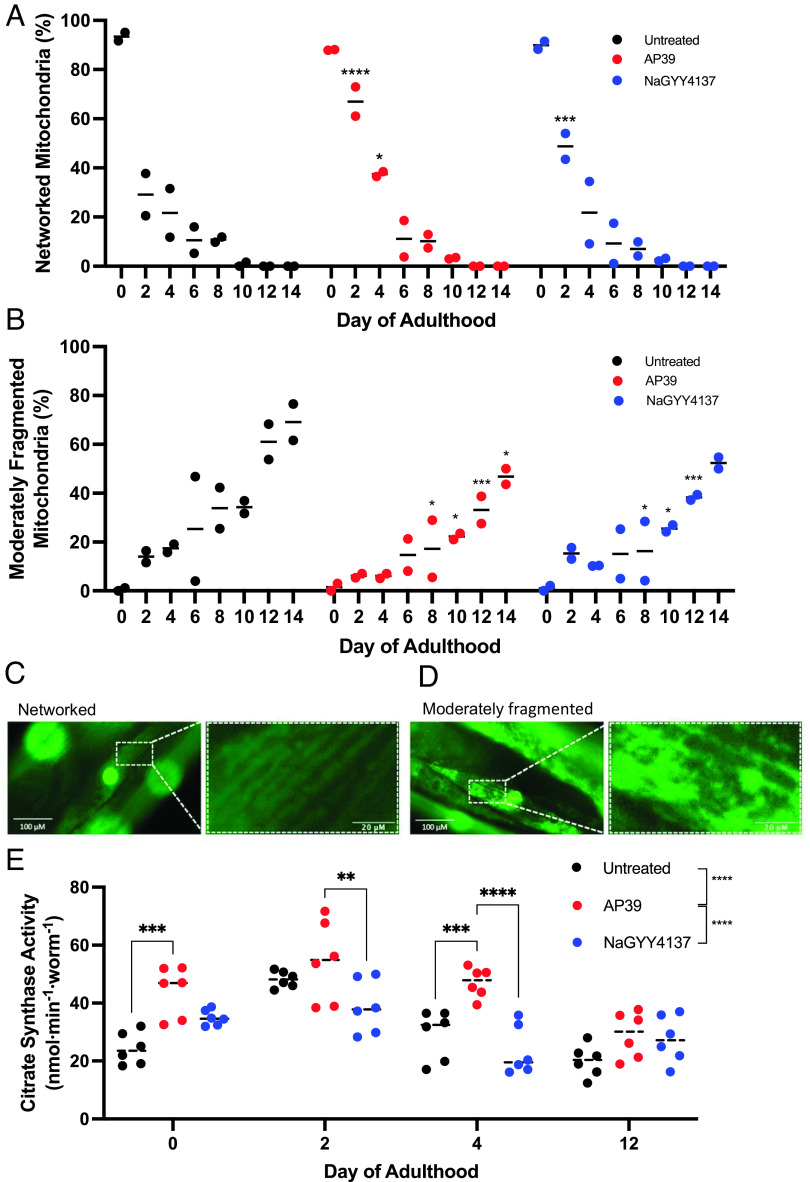
mtH_2_S prolongs mitochondrial integrity and content. (*A*) The percentage of well-networked and (*B*) moderately fragmented mitochondria during *C. elegans* aging is significantly improved with mtH_2_S (AP39), and for a longer duration than untargeted H_2_S (NaGYY4137) treatments. Data represent two biological replicates (total ~80 animals per time point/ condition and 450 muscle cells). (*C, D*) Representative green fluorescent protein-tagged mitochondrial images for normally arrayed (*Left*) and moderately fragmented (*Right*) mitochondria. White dashed boxes and corresponding magnified panels (*Right*) highlight each structural phenotype. (*E*) Citrate synthase activity with mtH_2_S at young adulthood (day 0) and day 4 postadulthood with mtH_2_S treatment, but not with untargeted H_2_S. Data represent two biological replicates, each with technical triplicates (total ~50 animals per time point/condition). All data are mean ± SD. *P < 0.05, **P < 0.01, ***P < 0.001, ****P < 0.0001 denote significant difference from untreated (0.01% DMSO) wild-type controls.

Given the well-established role of mitochondrial dysfunction in age-related health decline across species (25), we examined whether mtH_2_S health span promotion associated with maintained mitochondrial integrity. Using green fluorescent protein-tagged mitochondrion transgenic animals to compare lower dose mtH_2_S (100 nM) to higher dose untargeted H_2_S (100 µM), mitochondrial structure was scored as either well networked, or moderately fragmented. Well-networked mitochondria at day 0 of adulthood presented in 88% of wild-type animals, which was not affected by either mtH_2_S or untargeted H_2_S treatments. In line with previous reports (26), by day 2 postadulthood well-networked mitochondria reduced to 21% in wild-type worms. The number of well-networked mitochondria increased threefold with mtH_2_S treatment at day 2 of adulthood, and twofold with untargeted H_2_S (*P* < 0.001). Only mtH_2_S significantly sustained mitochondrial integrity at day 4 postadulthood (Fig. 3*A*). In wild-type animals, the number of moderately fragmented mitochondria increased progressively from day 2 of adulthood (21% of animals), reaching 86% by day 16. Comparable delays in moderate mitochondrial fragmentation were observed between mtH_2_S and untargeted H_2_S from days 8 to 12 postadulthood, whereas only mtH_2_S supressed moderate fragmentation up to day 14 of adulthood (Fig. 3*B*). We also assessed citrate synthase activity (CS) as a marker of mitochondrial health, which correlates with mitochondrial content, biosynthesis, and cristae area (43). Untargeted H_2_S failed to induce a significant effect on CS across the life course. Conversely, mtH_2_S significantly increased CS throughout life span, and to a greater extent than untargeted H_2_S (up to day 12, *P* < 0.0001), with significant increases in CS presenting up to day 4, but not day 12 of life span (Fig. 3*E*). To confirm that differences in H_2_S bioavailability does not underpin the improved efficacy of mtH_2_S vs. untargeted H_2_S for maintaining mitochondrial structure and content, we assessed total animal sulfide levels and found no difference with either compound at day 4 postadulthood (*SI Appendix*, Fig. S3). Thus, mtH_2_S improves mitochondrial integrity across age, which associates with health span maintenance.

### AP39-Mediated Health span Extension Requires H_2_S Metabolism and FoxO Pathways, but Not Nrf2 Oxidative Stress Protection.

Several mechanisms have been proposed to regulate longevity in response to exogenous H_2_S (19), yet the mechanisms governing health span extension are unknown. To probe this, we performed a hypothesis-driven RNAi gene knockdown and a mtH_2_S pharmacogenetic screen, using a microfluidic life span machine to assess animal health every day of the life course. First, we examined the requirement for enzymes controlling endogenous H_2_S synthesis: cytosolic cystathionine-β-synthase (CBS/*cbs-1*) and cystathionine-γ-lyase (CSE/*cth-2*), and cytoplasmic/mitochondrial 3-mercaptopyruvate sulfurtransferase (3-MST/*mpst-1*) (8–10). Corroborating previous reports, we found that *cth-2* knockdown alone had no effect on life span (19) and knocking down *cbs-1* or *mpst-*1 shortened life span (19, 44) (*SI Appendix*, Fig. S4). Knockdown of *cth-2* also did not significantly affect health span, whereas knockdown of *cbs-1* and *mpst-*1 RNAi both impaired health span (*SI Appendix*, Fig. S4). Cotreatment of RNAi against *cth-2*, *cbs-1* or *mpst-1* with mtH_2_S from L1 stage prevented the positive effects of mtH_2_S on life span and health span (Table 1 and *SI Appendix*, Fig. S4). While exogenous mtH_2_S might be anticipated to bypass endogenous H_2_S biosynthesis pathways, analysis of total animal sulfide levels confirmed a need for functional H_2_S producing enzymes, since mtH_2_S-induced sulfide increases in older age were ablated when combined with *cth-2*, *mpst-1* or *cbs-1* RNAi (*SI Appendix*, Fig. S5). Combined with the loss of mtH_2_S-induced life span and health span extension with knockdown of the H_2_S synthesizing enzymes *kri-1* and *cysl-2* (Table 1 and *SI Appendix*, Fig. S4), the H_2_S synthesis system is a general requirement for the positive effects of mtH_2_S donors on *C. elegans* health and longevity.

**Table 1.** Summary of mechanisms regulating mtH_2_S-induced life span and health span extension

	Gene target	Human Gene	Gene description	Required for positive effects of mtH_2_S?	
				Life span	Health span
H_2_S synthesis	*mpst-1*	MPST	Mitochondrial H_2_S synthesis	**✓**	**✓**
	*cth-2*	CTH	Mitochondria-translocating H_2_S synthesis	**✓**	**✓**
	*cbs-1*	CBSL	Cytosolic H_2_S synthesis	**✓**	**✓**
	*kri-1*	KRIT 1	H_2_S and ROS generation	**✓**	**✓**
	*cysl-2*	Cysteine synthase	Cytosolic H_2_S production	**✓**	**✓**
H_2_S oxidation/redox	*ethe-1*	ETHE1	Dioxygenase required for H_2_S oxidation	**✓**	**✓**
	*gsr-1*	GSR	Glutathione reductase	**✓**	**✓**
H_2_S + aging	*daf-16*	FoxO	H_2_S responsive FoxO transcription factor	**✓**	**✓**
Nrf2 oxidative stress protection	*skn-1*	NRF2	Regulates oxidate stress response	**✓**	**×**
	*gcs-1*	GCLC	Glutathione synthesis under Nrf2 control	**✓**	**×**
	*ikke-1*	RELA	Regulates Nrf2 nuclear translocation	**×**	**✓**
	*wdr-23*	Keap1	Negative regulator of Nrf2	**×**	**×**
H_2_S responsive	*hif-1*	HIF	H_2_S-responsive transcription factor	**×**	**×**
	*hsp-6*	Hsp70	H_2_S-responsive mitochondrial chaperone	**×**	**×**

*C. elegans* were treated with mtH_2_S (100 nM) from L1 larval stage in the presence or absence of RNAi against each target gene, using our microfluidic healthspan device. Each experiment was performed in duplicate (total ~160 animals per condition) and health span data expressed as area under the curve (% movement rate of each day of the lifecourse vs. day 0 baseline values). Ticks denote RNAi knockdown prevents significant (*P *< 0.05) mtH_2_S life span or health span extension and compared to untreated (0.01% DMSO) empty vector controls. Crosses denote RNAi knockdown does not prevent significant (*P* < 0.05) mtH_2_S-induced life span or health span extension. All raw data are provided in *SI Appendix*, Fig. S4.

We next examined the potential involvement of enzymes involved in wider H_2_S metabolism: ETHE1/*ethe-1*, a mitochondrial sulfur dioxygenase necessary for H_2_S catabolism (45), and GSR/*gsr-1*, a glutathione reductase involved in H_2_S-mediated production of glutathione (46). Knockdown of *ethe-1* alone did not affect life span but significantly increased health span and, when combined with mtH_2_S, prevented mtH_2_S-induced life span and health span extension. Because *ethe-1* catabolizes H_2_S, we postulated that harmful H_2_S accumulation might occur following combined exogenous mtH_2_S administration. Examining the dose response (1nM to 2 µM) of mtH_2_S + *ethe-1* knockdown revealed no further decline in animal health span, however life span became shortened at higher (100 nM to 2 µM) doses (*SI Appendix*, Fig. S6). Both life span and health span were reduced following *gsr-1* knockdown, which also inhibited life/health span extension with concurrent mtH_2_S treatment. Additionally, the FoxO/*daf-16* transcription factor has been implicated in H_2_S life span extension (19), and we observed lowered life span and health span with *daf-16* knockdown alone, corroborating previous reports (47). mtH_2_S did not increase life span or health span in animals subjected to *daf-16* RNAi (Table 1 and *SI Appendix*, Fig. S4).

H_2_S also regulated cellular redox homeostasis, in part through activation of the Nrf2 transcription factor and associated signaling pathway (48). We, therefore, knocked down Nrf2/*skn-1* or DCAF11/*wdr-23* as a negative upstream regulator of Nrf2 (49). In line with previous reports (50) *skn-1* knockdown animals were short lived, whereas *wdr-23* deficient worms were longer lived. Both *skn-1* and *wdr-23* RNAi also resulted in extended health span. Agreeing with earlier studies showing a need for the Nrf2 system for H_2_S-induced *C. elegans* life span extension (44), we also observed prevention of mtH_2_S increases in life span with combined *skn-1* or *wdr-23* knockdown. Despite this, our findings reveal that the Nrf2 system is not required for mtH_2_S-associated health span extension. We observed this same phenomenon with other components of the Nrf2 pathway, including the Nrf2-controlled glutamate–cysteine ligase catalytic subunit, GCLC/*gcs-1*. Only the Nrf2 nuclear translocation regulatory factor RelA/*ikke-1* attenuated mtH_2_S health span improvements (Table 1 and *SI Appendix*, Fig. S4). Our data strongly suggest that the positive health span effects of mtH_2_S were not dependent on the Nrf2 signaling system.

Last, we investigated the role of two H_2_S-responsive candidates, the mitochondrion-located heat shock protein chaperone HSPA9/*hsp-6* (44), and the hypoxia inducible transcription factor HIF1A/ *hif-1* (44). Knockdown of both *hsp-6* or *hif-1* significantly increased life span and health span, and combined gene knockdown with mtH_2_S did not impact the life span and health span promoting effects of mtH_2_S (Table 1 and *SI Appendix*, Fig. S4).

### Adult Treatments with mtH_2_S Extend Health span, but Not Life span.

**Fig. 4.**
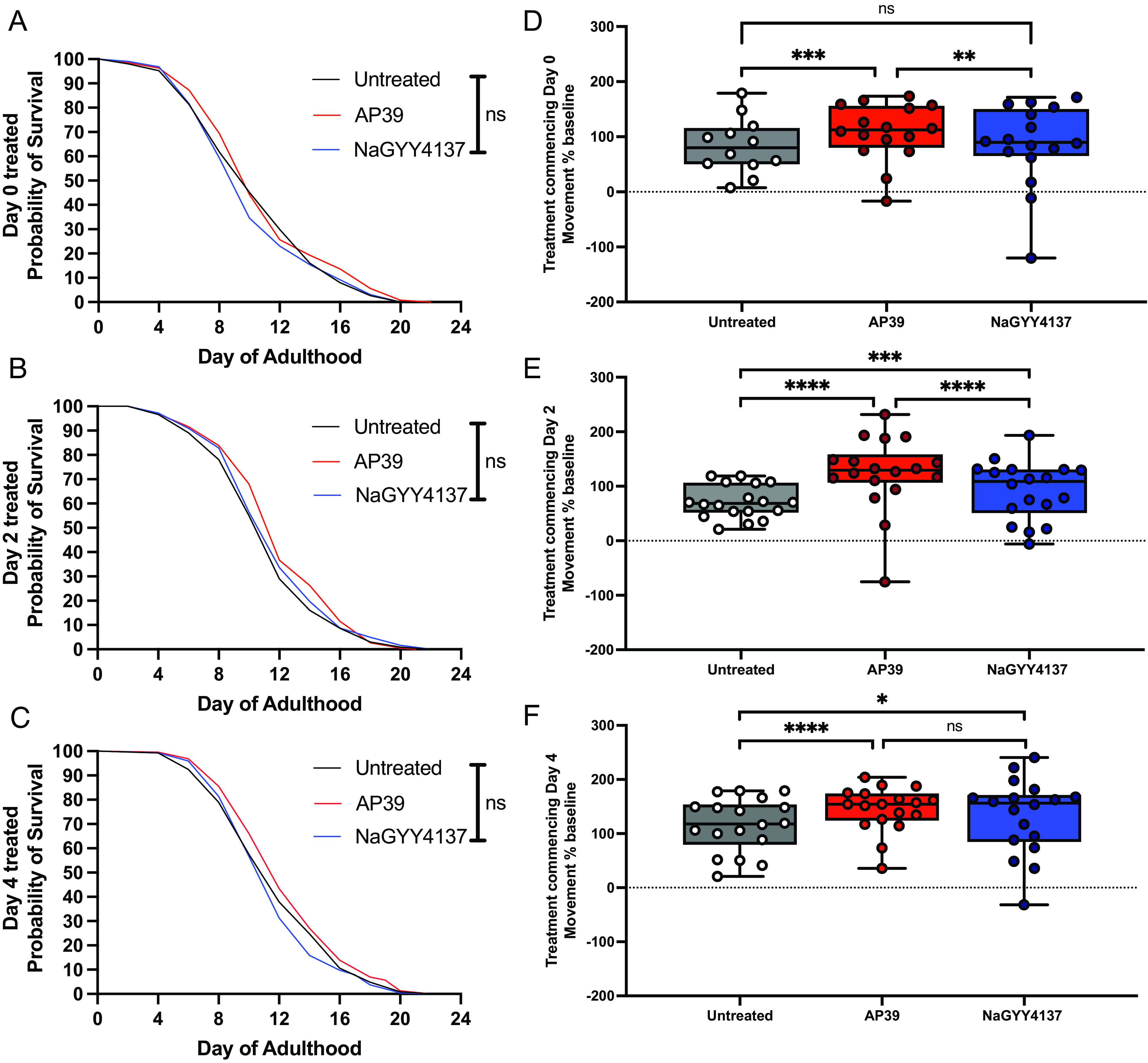
Adult-onset treatment with mitochondrion-targeted H_2_S extends health span but not life span. (*A*–*C*) Survival curves are unaffected vs. wild-type (*P* > 0.05) with 100 nM mtH_2_S and 100 µM untargeted H_2_S treatments beginning at day 0, 2 or 4 of adulthood. (*D*–*F*) mtH_2_S significantly increases health span when administered from day 0, 2, or 4 of adulthood, and untargeted H_2_S improves health span when administered from day 2 or 4 postadulthood. Health span data presented movement as a % change from day 0 baselines across all time points postadulthood, analyzed as area under the curve, n = 360 per condition, across 3 biological replicates and 18 technical replicates. Life span data are ~300 animals per condition, across three biological replicates. *P < 0.05, ***P < 0.001 and ****P < 0.0001) denotes significant difference vs. untreated (0.01% DMSO) wild-type controls.

Life span and health span extension have only been previously reported with untargeted H_2_S continuously administered from L1 larval stage until death. We, therefore, treated animals with low (100 nM) dose AP39 or 1000-fold higher (100 µM) dose NaGYY4137, starting in either day 0 young adults, day 2 of adulthood (chosen as the time point of mitochondrial fragmentation onset, Fig. 4*A* and ref. 26), or day 4 of adulthood [chosen as the time point when tissue structural integrity begins to decline (26)]. Both mtH_2_S and untargeted H_2_S donors were ineffective at increasing animal life span when administered from day 0, 2, or 4 of adulthood (*P* > 0.05). Conversely, health span was significantly increased by mtH_2_S when administered from either day 0, 2, or 4 of adulthood (*P* < 0.01) and was also increased with untargeted H_2_S treatments starting from days 2 or 4 postadulthood (*P* < 0.05), but not in day 0 young adults (Fig. 4 and *SI Appendix*, Fig. S7). Additionally, mtH_2_S administered from day 0 adulthood significantly increased the number of normally arrayed mitochondria at days 4 and 10 postadulthood, and improved sarcomere organization at day 10 postadulthood (*SI Appendix*, Fig. S8). Providing further evidence that improved mitochondrial health underpins mtH_2_S health improvements, we also found significantly lower mitochondrial superoxide levels during aging with adult mtH_2_S treatment (*SI Appendix*, Fig. S9). Health span was thus extended when mtH_2_S was delivered to adult animals, at time points where key aging subcellular defects occur, and is reflected in delayed onset of age-related dystrophic muscle and dysfunctional mitochondria.

### Adult mtH_2_S Treatments Maintain Mitochondrion- and Peroxisome-Enriched Transcriptomes in Later Life.

**Fig. 5.**
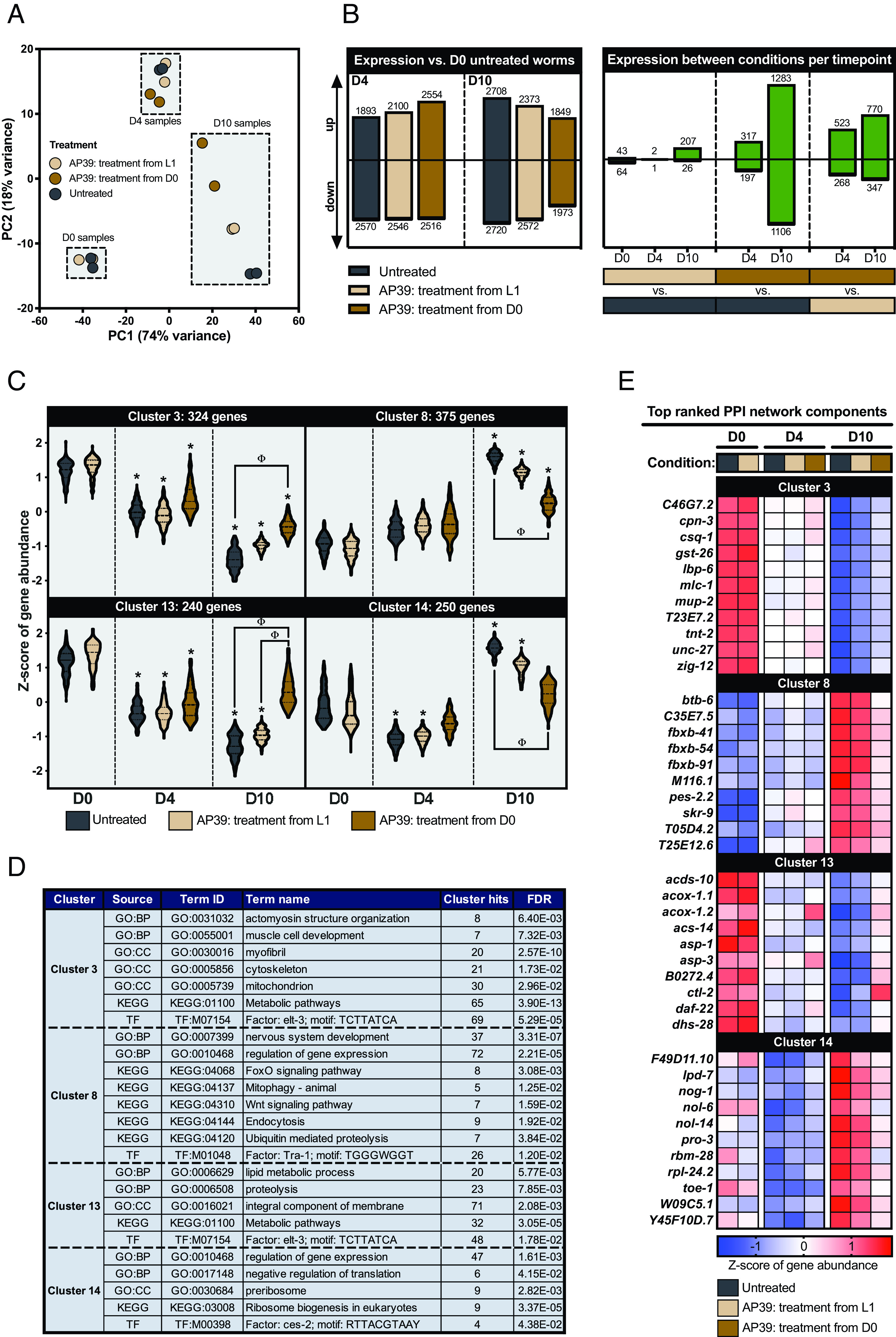
Effects of age and mtH_2_S on the *C. elegans* transcriptome. (*A*) Principal component (PC) analysis plot of all analyzed samples. (*B*) Differential gene quantities with time and between conditions. (*C*) Truncated violin plots depicting time/condition expression trends (represented as Z-score of gene abundance) for clusters of differentially expressed genes >200 genes in size. * = cluster genes have median FDR < 0.05 for given comparison with day 0 untreated (0.01% DMSO) wild-type animals, Φ = cluster genes have median FDR < 0.05 for direct comparison between treatments at given time point. (*D*) Representative term enrichments for each cluster shown in panel *C*. (*E*) Expression heatmap for top connected PPI network components for each gene cluster shown in panel *C*. Data represent ~60 animals across biological triplicates, per condition and time point. For all panels, WT = wild-type. D0, D4 and D10 = days 0, 2 and 4 postadulthood, respectively.

To better understand the molecules governing life span and health span responsiveness to mtH_2_S, we performed next-generation sequencing on animals treated with mtH_2_S from L1 larvae or from day 0 of adulthood. Principal component analysis revealed distinct features of *C. elegans* transcriptomes across days 0, 4, and 10 postadulthood. Moreover, mtH_2_S-treated animals displayed similar gene features to wild-type at day 0 and 4 of adulthood. Divergence from wild-type presented in older day 10 animals, with a shift toward day 4 features in mtH_2_S treatments, particularly when treated from day 0 young adulthood (Fig. 5*A*). Consistent with principal component analysis, global transcriptomic dysregulation at day 10 postadulthood was reduced in animals treated with mtH_2_S at adult onset only (Fig. 5*B*). Next, clustering of differentially expressed genes using expression profiles (51–53) identified four most prominent differentially expressed gene clusters (i.e., clusters containing >200 differentially expressed genes). Two of these (Clusters 8 and 14) exhibited an elevated expression profile in later life (day 10 postadulthood) that was suppressed with adult onset mtH_2_S but unaffected by mtH_2_S administered from birth (L1 stage), and were functionally associated with FoxO, proteolytic, mitophagy, and ribosome translational processes (Fig. 5 *C* and *D*). Analyzing the top 10 ranked protein–protein interaction (PPI) network hub nodes for each cluster identified *daf-2*-responsive F-box genes (*fbxb-41*, *fbxb-54*, *fbxb-91*, *M116.1*, *pes-2.2*, *T05D4.2*, *T25E12.6*) and autophagy (*C35E7.5*) components as prominent cluster 8 hubs. Cluster 14 hubs aligned almost exclusively to nucleolus localized components (*lpd-7*, *nol-6*, *nol-14*, *pro-3*, *rpl-24.2*) involved in RNA-binding activity as part of the ribonucleoprotein complex (*F49D11.10*, *rbm-28*, *toe-1*, *W09C5.1*, *Y45F10D.7*). As with wider cluster expression profiles, gene expression of hub components was predominantly altered at day 10 older age and in response to adult mtH_2_S treatments only (Fig. 5*E*).

Moreover, two gene clusters (Clusters 3 and 13) that aligned to cytoskeletal structure, mitochondria, and general metabolism functional classes, displayed progressive expression declines with age. In both clusters, mtH_2_S administered from L1 again failed to affect the age-related loss of gene expression. Conversely, adult-onset mtH_2_S treatment induced a strong maintenance of gene expression, emerging specifically in day 10 adults (Fig. 5 *C* and *D*). Additionally, this pattern of age-related gene expression changes remaining unaltered by L1 mtH_2_S administration, but rejuvenated by adult-onset mtH_2_S in later life emerged across nearly all other 27 gene clusters identified (*SI Appendix*, Table S1 and Fig. S10). Top ranked hubs for cluster 3 related mostly to muscle cytoskeletal proteins (*C46G7.2*, *cpn-3*, *mlc-1*, *mup-2*, *tnt-2*, *unc-27*), but included regulators of calcium homeostasis (*csq-1*) and glutathione transferase activity (*gst-26*). Within cluster 13, there was striking enrichment for hubs functionally associated with peroxisomal components (*acox-1.1*, *acox-1.2*, *B0272.4*, *ctl-2*, *daf-22*, *dhs-28*) and lysosomal cathepsin proteases (*asp-1*, *asp-3*). Again, only adult-onset mtH_2_S treatments appeared to attenuate the age-related reduction in gene expression of hub components, in older day 10 animals (Fig. 5*E*). To probe the mechanistic influence of these hub genes in the positive aging effects of adult-onset mtH_2_S, we examined the requirement of the peroxisomal catalase *ctl-2* as the most strongly maintained hub gene across clusters 3, 8, 13, and 14 (Fig. 5*E*). RNAi knockdown of *ctl-2* exacerbated early adult mitochondrial fragmentation and prevented the mtH_2_S-induced mitochondrial maintainance in early and later life (*SI Appendix*, Fig. S11), therein supporting the functional and mechanistic relevance of our identified transcriptomic targets.

### A GATA Transcription Factor Regulatory Circuit Underpins the Positive Aging Effects of mtH_2_S.

**Fig. 6.**
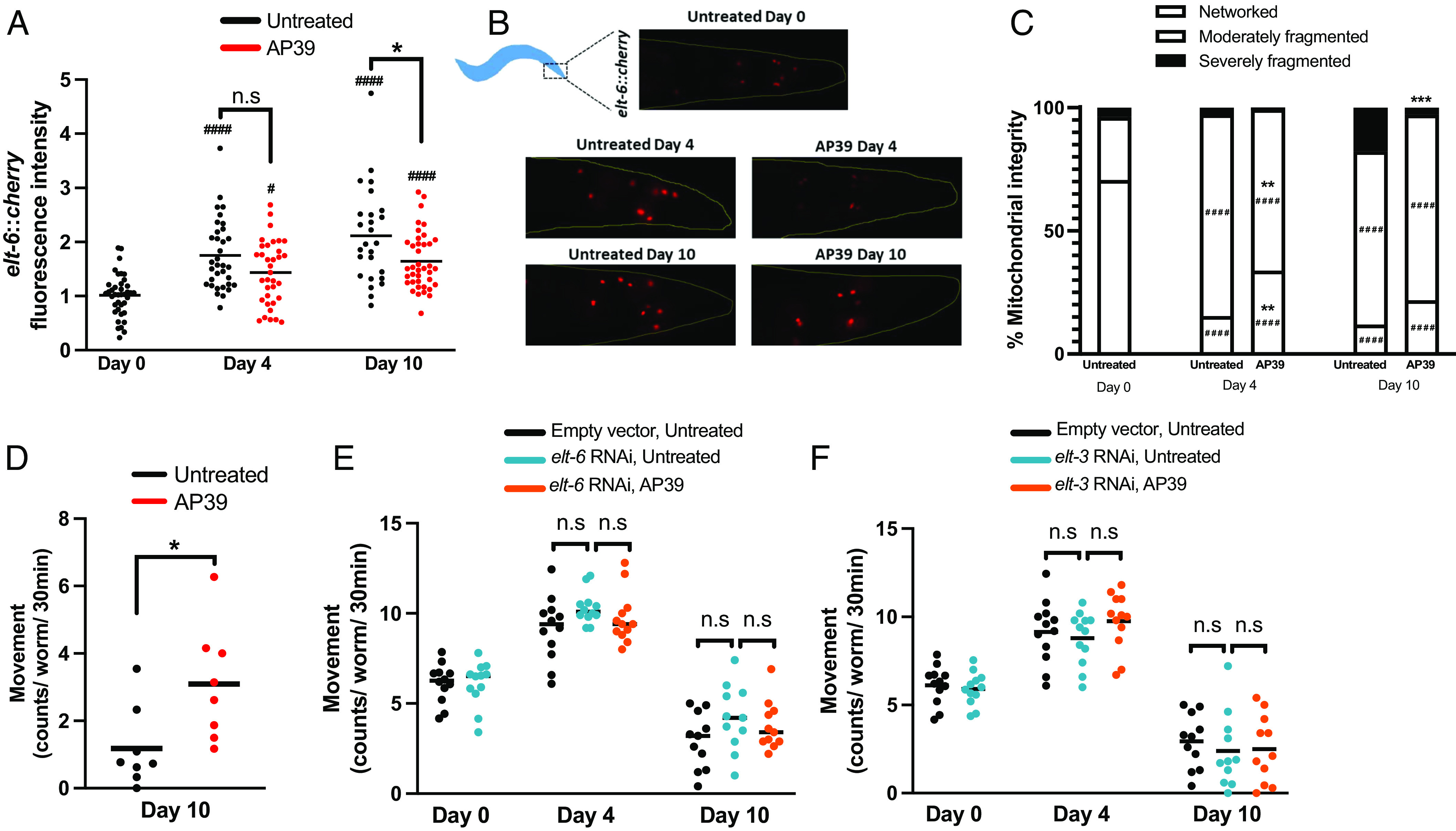
Adult-onset mtH_2_S preserves health span through the ELT*-6* GATA transcription factor circuit. (*A*) ELT-6 expression increases with aging and is significantly repressed with AP39 treatment compared to untreated animals in later-life. (*B*) Representative images of ELT-*6* expression (*elt-6::mCherry*). (*C*) Preservation of age-related declines in mitochondrial integrity by AP39 correspond with attenuated ELT-6 expression (using *elt-6::mCherry* + *mito::*GFP coexpression reporter strain). (*D*) AP39-induced improvements in aging movement capacity is confirmed in *elt-6::mCherry* + *mito::*GFP animals, and correspond with lowered ELT-6 and improved mitochondrial integrity. RNAi against *elt-3* (*E*) and *elt-6* (*F*) prevents the health span–promoting effects of AP39. Panels *A*–*D* employed transgenic animals coexpressing *elt-6::mCherry* + *mito*::GFP in body-wall muscle, across 25 to 45 animals and two biological repeats. Panels (*E* and *F*) employed wild-type N2 animals. Movement rates are from 80-120 animals per condition, per time point. # denote significant effect of aging compared to untreated day 0 animals (^#^*P* < 0.05; ^###^*P* < 0.001). * denote significant effect of treatment for within-day comparisons against untreated animals (*P < 0.05; **P < 0.01; ***P < 0.001).

We next sought to examine the mechanistic role of genes responsive to mtH_2_S during aging; however, the clusters identified represent several dozen individual differentially expressed genes. We, therefore, employed transcription factor (TF)-binding site analysis to identify TFs predicted to commonly regulate the cytoskeletal (Cluster 3) and peroxisomal (Cluster 13) gene clusters that display mtH_2_S-induced preservation in older age. From this, a single transcription factor, *elt-3* (part of a GATA family of transcription factors), emerged as the putative regulator of both gene clusters (Fig. 5*D*). During wild-type aging, expression of *elt-5* and *elt-6* increase which, in turn, repress expression of *elt-3* to regulate a large portion of age-related transcriptome changes (54). Our untreated controls mirrored this response at the transcriptional level, and these expression changes are reversed by adult onset mtH_2_S (*SI Appendix*, Fig. S12). Next, using transgenic animals coexpressing ELT-6 RFP and mitochondrial GFP reporters, we confirmed protein level ELT-6 upregulation during aging, which was suppressed by adult onset mtH_2_S treatment (Fig. 6 *A* and *B*), and corresponded with improved mitochondrial structure and movement rates (Fig. 6 *C* and *D*). Additionally, knockdown of either *elt-6* or *elt-3*, while not affecting animal movement rate in middle age and older age, prevented mtH_2_S-induced movement increases (Fig. 6 *E* and *F*).

To verify the role of *elt-6*/*elt-3*, we examined the cytoskeleton (adherens junction) and mitochondrion-localized protein BAR-1/ β-catenin, loss of which is reported to upr-egulate gene clusters under regulation by *elt-3* (55) which would, therefore, be anticipated to also down-regulate ELT-6. Consistent with this model, we observed that *bar-1* RNAi prevented age-related increases in ELT-6 expression (*SI Appendix*, Fig. S13). Moreover, mtH_2_S did not synergistically lower age-related ELT-6 levels when combined with *bar-1* RNAi (*SI Appendix*, Fig. S13), implying some functional association with mtH_2_S and bar-1/β-catenin (further supporting a role for mtH_2_S in modifiying the cytoskeleton via the *elt-6*/*elt-3* circuit). While the precise causal mechanisms linking H_2_S-related mitochondrial improvements with the *elt-6*/*elt-3* system and, subsequently, health span remains undefined, the mitochondrial mechanisms appear specific to the aging context; although we show aging mitochondrial decline and mtH_2_S acts through this TF circtuit, inducing acute severe mitochondrial decline via toxic drugs fails to activate ELT-6 despite major structural fragmentation of mitochondria (*SI Appendix*, Fig. S14). Combined, our systems biological studies provide evidence that mtH_2_S improves mitochondrial health to alter *elt-6*/*elt-3* TFs, which likely act as a reguatory circuit governing cytoskeletal and peroxisomal gene clusters to, ultimately, modify health span.

## Discussion

H_2_S is a diatomic signaling molecule that promotes healthy aging in *C. elegans* (20), yet the underlying mechanisms and therapeutic viability across the lifecourse remains unclear. In this study, we have demonstrated that low mtH_2_S doses extend *C. elegans* health span, which associates with improved mitochondrial integrity from young adulthood into older age. Multiple elements of H_2_S metabolic pathways, and FoxO transciption factors emerged as mechanisms governing both life span and health span, whereas the Nrf2 antioxidant system is dispensible for mtH_2_S-induced health span extension. Adult mtH_2_S treatments also increase health span, predominantly in later life, and associates with rejuvenation of key features of the aging transciptome, including mitochondrial function, cytoskeletal content, and peroxisomal metabolism, which appear to be controlled by a GATA transcription factor circuit.

The ability of large amounts of untargeted H_2_S, administered from birth, to enhance *C. elegans* life span and health span, is well documented (17–20). Our data reveal 1000-fold lower doses of a mitochondrion-targeted (TPP^+^-driven) H_2_S donor (28) can account for the life span, health span, and neuromuscular strength extension elicited by H_2_S. Thus, small amounts of H_2_S transported to the mitochondria are likely responsible for, and the site of action of, H_2_S effects on longevity. Temporal analysis further revealed mtH_2_S improved mitochondrion integrity beginning in earlier life that was maintained throughout the lifecourse, thus delaying one of the primary cellular hallmarks of aging (25). Conversely, mtH_2_S did not increase movement rates or muscle strength until older age, likely owing to a lack of declines in muscle strength and habitual movement capacity during early adulthood, which is unsurprising, if decreasing H_2_S metabolism/synthesis is an aging pathology (56). These findings closely mirror the human phenotype, where a clear biphasic pattern of muscle aging emerges that involves early disruption to metabolic processes (57, 58) (as with *C. elegans* early loss of mitochondria integrity), which later manifests as exponential neuromuscular strength/ physical capacity declines (again, as occurs in *C. elegans*) that exceeds rates of musle mass losses (59, 60). As such, our data evidence that mtH_2_S can target the early mitochondrial metabolic perturbations during aging, possibly in preference over targeting respiratory function of existing mitochondria (61, 62) that attenuates the ensuing later changes in neuromuscular performance and health (25, 61, 62).

While the mechanisms regulating H_2_S-induced longevity have been explored (19), understanding the molecules governing health span is at least equally valuable, given the growing societal burden of life span – health span dissociation. Of the fourteen genes targeted for established roles in H_2_S biology, two clear functional themes emerged as mechanisms of mtH_2_S life span and health span extension. First, the FoxO/*daf-16* transcription factor is a highly conserved regulator of longevity across species (63) and in response to untargeted H_2_S (19). Our findings extend this to show *daf-16* is also required for mtH_2_S-related health span improvements. Second, although exogenous H_2_S could hypothetically bypass the biochemical need for endogenous H_2_S synthesis, the H_2_S metabolism genes examined (most strikingly the mitochondria localized 3-MST/*mpst-1*) were required for health span extension by mtH_2_S. Moreover, mtH_2_S-induced sulfide increases were prevented by *cth-2*, *mpst-1,* or *cbs-1* knockdown. Thus, while perhaps counterintuitive, presence of a functional H_2_S production system is a requirement for efficacious mtH_2_S treatments and might reflect the multifaceted cellular roles of the H_2_S enzymatic machinery and/or the diverse downstream consequences of loss of these enzymes (64). For example, H_2_S likely exerts at least part of its biological effects via cysteine persulfidation of multiple protein targets (65). The mitochondrially localized 3-MST/*mpst-1* is also a trans-persulfidase (66), thus H_2_S enzyme knockdown could prevent mtH_2_S-mediated persulfidation events, through which H_2_S might partially act. Interestingly, despite fatal consqeuences of complete ETHE1 knockout in higher mammals (45), RNAi knockdown of the mitochondrial H_2_S catabolic enzyme ETHE1/*ethe-1* strongly improved health span, possibly by mimicking mtH_2_S through mitochondrial accumulation of noncatabolized endogenous H_2_S. However, combined *ethe-1* RNAi and mtH_2_S ablates health span extension, and life span becomes shortened at higher, but not lower (1 to 10 nM) mtH_2_S doses, potentially due to toxic mtH_2_S accumulation, implying tight physiological range of mtH_2_S hormesis. Overall, these mechanistic insights add further evidence that place mitochondria at the center of H_2_S-regulated health span.

Activation of the Nrf2/*skn-1* antioxidant system has also been reported to control H_2_S-based longevity in *C. elegans* (48). Oxidative stress and reactive oxidant species might be a secondary consequence of tissue aging that may exacerbate, rather than cause aging health decline (67–70). While corroborating the requirement of the Nrf2 pathway for H_2_S life span extension, multiple components of the Nrf2/*skn-1* system, including the upstream activator DACF11/*wdr-23* and downstream effector GCLC/*gcs-1*, were not required for mtH_2_S health span improvements. Knocking down KRIT1/*kri-1*, which activates Nrf2 through redox species generation (44), did prevent mtH_2_S-induced health span extension. However, since several Nrf2 system components are not mechanisms of mtH_2_S health span extension, KRIT1/*kri-1* health span regulation likely relies on its alternate functions in H_2_S synthesis (44). Importantly, these results highlight clear dissociation between the fundamental mechanisms governing life span vs. health span, indicating that oxidative stress protection pathways are dispensable for mtH_2_S-extended health span, but not life span.

The potential for developmentally programmed metabolic patterns with larval H_2_S treatments (21–23) renders the efficacy of postadulthood H_2_S therapies uncertain. We establish health span alone is extended with young adult mtH_2_S treatments, and when initiated in the presence of existing aging tissue pathologies. We, and others (26), found that mitochondrial fragmentation begins early in life (2 days postadulthood), and in this study we report that mtH_2_S improves health span when administered at this time. Similarly, muscle structural and proteostasis abnormalities occur later (4 days postadulthood), and mtH_2_S also displays efficacy during this therapeutic window. Mitochondrial H_2_S is, therefore, a viable adult-onset antiaging therapeutic opportunity. Transcriptomic studies aimed at understanding the mechanisms regulating adult mtH_2_S health span extension revealed clear distinctions from larval treatments. Whilet administering mtH_2_S from L1 stage caused broad transcriptional features that diverged only slightly from wild-type animals only in older age, adult-onset mtH_2_S caused strong rejuvenation of the aging transcriptome toward “younger” gene profiles. Cluster analysis revealed adult mtH_2_S suppressed aging-induced increases in FoxO/*daf-16* pathway expression. Whilet contrasting our observation that *daf-16* is a required effector of larval mtH_2_S treatment, suppression of aging *daf-16* levels with adult mtH_2_S implies divergent effects of FoxO induction during development vs. postdevelopment (71). This is consistent with earlier reports that longevity caused by up-regulating FoxO/*daf-16* (*via* insulin receptor/*daf-2* mutation) is primarily established during larval development (72). Conversely, sarcopenia associates with postdevelopmental FoxO upregulation across species (58), corresponding with later life metabolic reprogramming to compensate for aging health decline (57, 58). Consistent with a potential beneficial aging effect of reduced postadulthood FoxO expression, are the moderate health span improvements we report with mtH_2_S-related *daf-16* suppression. This phenomenon underscores an unexplained paradox in aging research, whereby developmentally programmed life span extension (e.g., with increased FoxO signaling) presents at the expense of health span (7). Indeed, in people, FoxO genetic variants correlate with centenarians (73), yet impaired insulin signaling/ increased FoxO induced during adulthood causes serious clinical complications (e.g., diabetes, sarcopenia). Our data support a model whereby postdevelopmental, aging-induced increases in FoxO expression are harmful to health span (71), and drug interventions such as mtH_2_S that lower this response improve health whilet having minimal effect on longevity. Last, adult mtH_2_S also caused suppression of age-related increases in mitophagy and, most prominently, ribosomal biogenesis. Thus, mtH_2_S might mitigate unchecked mitophagy by preventing the mitochondrial dysfunction that promotes mitophagy with age (74), and promote improved translational efficiency as previously proposed to underpin physical activity-based anti-aging regimens (75, 76).

Adult mtH_2_S treatment also better maintained later-life expression of lowered aging transcriptomic profiles. Genes clustering to mitochondrion function featured heavily, lending further support to the central role of mitochondria in mtH_2_S-mediated health span. Expression of muscle cytoskeletal components also decreased with age, which was minimized by adult mtH_2_S administration, with top ranked hub components largely represented by cytoskeletal (e.g., troponin regulation) factors. Given the later life temporal correlation between increased muscle structural genes and movement/strength capacity, mtH_2_S represents a promising neuromuscular health intervention in older age. Last, loss of metabolic plasticity is a common feature of aging across species (25). We found progressive reduction in expression of metabolic functional clusters across aging that was increased with adult onset mtH_2_S. Notably, metabolic cluster hub genes were strongly enriched for peroxisomal components. Peroxisomes are essential for proper functioning of all cell types, compartmentalizing enzymes regulating, e.g., fatty acid β-oxidation and hydrogen peroxide metabolism. Several facets of peroxisome dysfunction cause accelerated aging (77), and our data suggest peroxisome function can be sustained by mtH_2_S across life span. For example, *dhs-28* regulates age-dependent peroxisome loss, knockdown of which extends *C. elegans* life span (78), and our findings identify *dhs-28* as a top ranked, mtH_2_S-responsive hub component. Moreover, we confirm the mechanistic relevance of our identified peroxisomal transcriptomic targets by showing knockdown of the *ctl-2* hub gene prevents mtH_2_S-induced health span extension. Growing evidence also highlights inextricable cross talk between peroxisome and mitochondrial function (79). Indeed, caloric restriction–induced longevity [whose mechanisms converge with those of H_2_S (80)], requires mitochondria structural maintenance which, in turn, promotes peroxisome fatty acid oxidation (81). Our early-life improvements in mitochondrial integrity, combined with mitochondria localizing H_2_S, demonstrate improvements in mitochondrial integrity precede, and perhaps regulate, later life improvements in peroxisome capacity. Additionally, the lack of mechanistic regulation of health span by the Nrf2 antioxidant system implies that improved peroxisome fatty acid oxidation, as opposed to lowered reactive oxygen species generation, underpin peroxisomal effects of mtH_2_S on healthy aging.

Transcription factor-binding site analysis of our transcriptomic data identified the GATA TF circuit *elt-6*/*elt-3* as putatively regulating the health span benefits of adult onset mtH_2_S, which were subsequently verified by our mechanistic experiments. Previous work established age-related *elt-6* upregulation and downstream repression of *elt-3*, which accounted for altered expression of ~1,300 “aging” genes (54), which might comprise an evolutionarily conserved element of natural aging adaptation that promotes longevity, perhaps at the expense of health span, similarly to FoxO. Here, we confirm the relevance of this aging TF axis and establish *elt-6*/*elt-3* as mechanisms through which mtH_2_S and, either interrelatedly or independently *via bar-1*/ β-catenin, act to affect aging health. Interestingly, mitochondrial dysfunction per se is insufficient to explain *elt-6* upregulation, since severe toxin-induced mitochondrial insults fail to induce ELT-6. Thus, some as yet unknown specificity to age-related mitochondrial decline alters the *elt-6* pathway to induce gene expression changes that impair health span. Regardless, our combined functional, morphologic, transcriptomic, and mechanistic results point to a model where aging mitcohondrial decline activates GATA TFs to alter gene expression, centered on cytoskeletal and peroxisomal gene clusters, to impair animal health. Crucially, mtH_2_S is an efficacious therapeutic approach for targeting the mitochondria to reverse this aging signaling axis and, ultimately, improve health span.

In conclusion, a systems biological approach identifies the mitochondria as the primary site of H_2_S action for slowing aging, with distinct molecular mechanisms underpinning life span vs. health span extension. Unlike life span, increased mtH_2_S health span does not require activation of the Nrf2 antioxidant system. Adult-onset mtH_2_S also increases health span alone, which associates with unique aging transcriptomic signatures compared to life span–extending developmental mtH_2_S treatments, under the control of *elt-6*/*elt-3* GATA transcription factors. The emergence of neuromuscular health improvements in later life might also be underpinned by temporally correlated, mtH_2_S-responsive transcriptomic features of mitochondria, peroxisomal metabolism, and cytoskeletal function. Finally, the comparably lower mtH_2_S donor doses (vs. nontargeted NaGYY4137; >3 orders of magnitude difference) required for aging health benefits, combined with efficacy in adult animals and high conservation of associated mechanisms, renders mtH_2_S a possible translational antiaging therapy.

## Materials and Methods

### *C. elegans* Maintenance and Experiment Design.

The strains used in this study were N2 wild-type and CB5600 *(ccIs4251 (Pmyo-3::Ngfp-lacZ; Pmyo-3::Mtgfp))* and were obtained from the Caenorhabditis Genetics Centre (CGC, University of Minnesota). For maintenance, *C. elegans* were cultured at 20 °C on OP50 *E. coli* seeded NGM agar plates, as previously described (82). For all experiments, the first day of adulthood was considered as Day 0.

For drug exposure experiments, unless stated otherwise, L1 worms synchronized by gravity floatation were cultured at 20 °C on OP50 *E. coli* seeded NGM plates containing either 100 nM AP39 + 0.01% DMSO, 100 nM or 100 mM NaGYY4137, 0.01% DMSO or no drug. The mitochondrion-targeted H_2_S donor compound, AP39, was synthesized in-house by the Whiteman lab, as previously described (83, 84). The NaGYY4137 (85) untargeted H_2_S donor compound was also synthesized in-house. Compound solutions were freshly prepared for every use and added to plates the evening before animal transfers. For developmental treatments, drug dosing was started from the L1 larval stage and continued throughout the life course. For adult drug treatments, gravity synchronized L1 larvae were grown on NGM agar seeded with OP50 only for 60 h to reach young adulthood, after which drug treatments were started at either day 0, day 2, or day 4 postadulthood. Adult animals were transferred every 48 h to fresh plates to remove progeny and maintain consistent food and drug concentrations.

### Survival Assay and Measures of *C. elegans* Locomotion and Maximal Strength Production.

Adult animals were scored and transferred to fresh plates every 48 h. The animals were scored as dead when they failed to move in response to stimulus with a needle. Animals that were lost, killed during transfer, or died as a result of (e.g.) egg laying defects were censored. Total animal numbers were n = 300 per condition, across three biological replicates. Locomotion assays were performed using WMicrotracker and maximal strength production was assessed using the ‘NemaFlex’ microfluidic device (36) (See *SI Appendix, Supplemental Methods*).

### Mitochondrial and Myofibrillar Imaging.

Mitochondria within body wall muscle cells of the CB5600 (*ccIs4251* (*Pmyo-3::Ngfp-lacZ; Pmyo-3::Mtgfp*)) strain were imaged using an Olympus CKX41 microscope (Olympus UK Ltd. London). The worms were imaged by GFP fluorescence microscopy at 40× magnification. Approximately 20 to 30 animals per condition were placed in 20 µL M9 on a microscope slide and immobilized with a cover slip. Images were taken of myofibers or mitochondria in body-wall muscle from both head and tail regions of every animal and visually classified as either well-networked, moderately fragmented, or severely fragmented (for mitochondrial quantification), or organized, moderately disorganized, and severely disorganized (for myofibrillar quantification) as previously described (26, 86). The overall proportion of mitochondrial or myofibrillar classifications was obtained by normalizing to the total muscle cell count within each treatment condition (~150 to 300 muscle cells per condition from 30 to 60 animals per time point) across two biological replicates.

### Measurement of Citrate Synthase Activity.

Wild-type N2 animals were roughly age synchronized as previously described and grown for ~60 h to young adulthood on fresh OP50 bacterial lawns 20 °C. Animals were transferred to fresh OP50 plates every 48 h to remove progeny and prevent population starvation, and 50 animals collected per condition, per time point (days 0, 2, 4, and 12 postadulthood) and per replicate. Citrate synthase activity (CS) was measured in isolated mitochondrial pellets, as described in Supplemental methods section.

### RNA Interference Protocols.

All RNAi experiments were performed using age synchronized L1 larval stage animals by gravity flotation and grown for 60 h on NGM agar plates containing 1 mM IPTG, 50 µg/mL ampicillin. The plates were seeded with 200 μL HT115 (DE3) bacteria expressing double-stranded RNA against the genes screened. The Ahringer RNAi library (87) was utilized, purchased from Source Bioscience (Cambridge, UK). HT115 (DE3) bacteria containing the empty L4440 plasmid vector was used as controls. Full details of each RNAi protocol for health span/life span screens, or plate-based experiments can be viewed in *SI Appendix*, *Supplemental Methods* section.

### RNA Isolation for Next-Generation Sequencing.

Synchronized worms were grown on NGM agar plates until young adulthood and treated with AP39 mtH_2_S from either L1 or day 0, as described above. On sample collection day, 100 worms were manually picked and added to 1 mL TRIzol™ Reagent (Thermofisher Scientific, Loughborough, UK) prior to RNA extractions (see *SI Appendix*, *Supplemental Methods* section for details).

### RNA-seq Data Analyses.

After RNA sequencing data preprocessing (see *SI Appendix*, *Supplemental Methods* section), the DESeq2 package for R (88) was used to test for differential gene expression. Establishing approaches to adaptive shrinkage methods (89) and control for false discovery rate (FDR) were employed. DEGreport R was applied to normalized counts to group by expression profile any gene differentially regulated between treatments and/or time points. Functional enrichment analysis of defined gene clusters was then undertaken using the gprofiler2 package for R (90). Each gene cluster was also input into the Online Search Tool for Retrieval of Interacting Genes/Proteins (STRING, v11.5; ref. 91) to infer respective PPI networks. Full details of the bioinformatic pipeline employed are provided in Supplemental methods section.

### 7-azido-4-Methylcoumarin Measures of Total Sulfide Levels.

In all, 120 animals were picked into M9 buffer in 1.5 mL low-bind eppendorphs and washed three times with 1 mL sterile M9 to clear bacterial debris and progeny. Samples were snap-frozen in 40 µL M9 and stored at −80 °C until analysis (within 1 wk). Sulfide content was assessed as described previously (13, 86) as described in *SI Appendix*, *Supplemental Methods* section.

### ELT-6 Fluorescent Reporter Quantification.

The SD1550 strain harboring an *elt-6* fluorescent promoter and mitochondrial GFP (*ccIs4251 [(pSAK2) myo-3p::GFP::LacZ::NLS + (pSAK4) myo-3p*::mitochondrial GFP + *dpy-20*(+)] I. *stIs10178* [*elt-6p::HIS-24::mCherry + unc-119*(+)] was imaged on an upright epifluorescent microscope (BX43, Olympus Life Science, UK). All *elt-6* images were taken with *mCherry* fluorescence at 500-ms exposure and GFP fluorescence set to 50-ms exposure for mitochondrial images. Single images were taken from the head for ELT-6, as the sole site of ELT-6 reporter expression pattern, and from head and tail regions for mitochondrial characterisation across 40-60 animals per condition, per time point. Mitochondrial images were analyzed as detailed above, and ELT-6 images were quantified in ImageJ by performing integrated density quantification on each fluorescent image, subtracting background fluorescence.

### Mitochondrial Toxic Stress Assays in ELT-6 Transgenic Reporter Animals.

Age-synchronized SD1550 animals were grown to young adulthood on 33-mm NGM plates seeded with OP50 bacteria. Approximately 30 day 0 adults where then picked into 40 μL of either 100 μM hydrogen peroxide (Sigma, UK; 7722-84-1) or 5 mM sodium arsenite (Santa Cruz, DE; 7784-46-5) diluted in M9 for 1- and 2-h exposures, respectively. For rotenone and antimycin A exposures, animals were picked into 40 μL 50 μM or 100 μM concentrations, respectively, diluted in 20 mg/mL OP50/NGM bacteria and incubated for 4 h. Tubes where then washed three times with M9 before animals were pipetted onto slides and immobilized by a coverslip. Both ELT-6 and mitochondrial images were then captured and analyzed as detailed above.

### Mitochondrial Superoxide Assessment.

Superoxide levels were measured using MitoSOX (Invitrogen, UK) as described previously (86, 92). For day 0 measures, animals were grown on OP50 plates until L3 larval stage, where ~30 animals were then picked onto petri plates + OP50 seeded with a final plate concentration of 10 μM MitoSOX and left to incubate in the dark for 24 h. The same exposures were performed for day 4 and day 10 measures (i.e., day 3 and day 9 adults picked onto MitoSOX plates for 24 h, respectively). On the day of imaging, animals were washed from plates with M9 buffer into 1.5 mL low-bind tubes and washed three times to clear the outer cuticle of probe. Animals were then placed on OP50 plates for 1-h to clear residual probe from the gut. Animals were then picked into 20 μL M9 buffer on glass slides with glass coverslip. Images were taken with a 40× objective with green light excitation and a 1-s exposure rate. The terminal bulb was manually selected in ImageJ and integrated fluorescence density was normalized to the total area of analysis and background fluorescence removed.

### Statistics and Data Analysis.

Statistics and graph generation were performed in GraphPad Prism 9. Significance was determined by paired *t* test or 2-way ANOVA, with post hoc multiple comparison tests. For survival analyses, the Log-Rank (Mantel Cox) test was used.

## Supplementary Material

Appendix 01 (PDF)

Dataset S01 (XLSX)

## Data Availability

All study data are included in the article and/or supporting information. The raw RNA sequencing data can be found within the NCBI BioProject database (https://www.ncbi.nlm.nih.gov/bioproject/) under the Sequence Read Archive (SRA) accession PRJNA996496 (93).
