# Supplementary material for: Mitochondrial sulfide promotes life span and health span through distinct mechanisms in developing versus adult treated Caenorhabditis elegans
Source: Proc Natl Acad Sci U S A. 2023 Jul 31;120(32):e2216141120. doi: 10.1073/pnas.2216141120 (PMC10410709; doi:10.1073/pnas.2216141120)
Supplement: Supplementary file 1 — Appendix 01 (PDF) [file pnas.2216141120.sapp.pdf]

PNAS

Supporting Information for
Mitochondrial sulfide promotes lifespan and healthspan through distinct mechanisms in developing *versus* adult treated *Caenorhabditis elegans*

Adriana Raluca Vintila[1*], Luke Slade[1,2*], Michael Cooke[1,3*], Craig R. G. Willis[4], Roberta Torregrossa[2], Mizanur Rahman[5], Taslim Anupom[6], Siva A. Vanapalli[5], Christopher J Gaffney[7], Nima Gharahdaghi[2], Csaba Szabo[8], Nathaniel J. Szewczyk[3,9], Matthew Whiteman[2†], Timothy Etheridge[1†].

* denotes equal first authorship.

Dr Timothy Etheridge, Department of Public Health and Sports Sciences, Faculty of Health Life Sciences, University of Exeter, St. Luke's Campus, EX1 2LU, UK.

Email: t.etheridge@exeter.ac.uk

This PDF file includes:

Supplemental Methods
Supplemental Figures 1 to 14
Legend for Supplemental Table 1

Other supporting materials for this manuscript include the following:

Supplemental Table 1

Supplemental Methods

WMicroTracker locomotion assay

Locomotion was measured at days 0, 2, 4, 8, 12 and 16 post-adulthood, using the WMicroTracker One (Phylumtech, S.A. Santa Fe, Argentina). Worms were collected from NGM agar plates and added to 100 µL of M9, in a 96 well flat bottom plate. Animal movement was measured over 30 minutes and normalised to counts per worm. The data is presented as an average of 3 biological replicates, each with 6 technical replicates of 20 worms per well for a total of n=120 per condition, per time point.

Measurement of maximal C. elegans strength production

C. elegans muscle strength was assayed using the NemaFlex microfluidic device which involves deflection of soft microfabricated pillars by moving worms, as previously published by our laboratory (1). For days 0, 2, 4 6, 8 and 10 post-adulthood, animals were pipetted into the force-measurement device, and images were recorded at 5 frames/s for 0.5 min for each worm. Pillar displacement was measured using a custom pillar deflection tracking code written in Matlab (R2013b; Mathworks, Natick, MA, USA), and converted into the corresponding forces using a modified form of the Timoshenko beam deflection theory. The maximal forces from each frame were binned to build a cumulative force distribution. Animal strength was defined as the 95th percentile of this maximal force distribution. At least 15 animals per condition and per time point were used to generate population maximal strength values. All experiments utilised age synchronous animals at 20 ± 1°C.

Measurement of citrate synthase activity

Samples were washed from plates using M9 buffer and washed again using centrifugation. The final wash was completed with homogenization buffer (100 mM KCl, 50 mM KH_2PO_4, 50 mM Tris, 5 mM $MgCl_2$, 1 mM EDTA, and 1.8 mM ATP at pH 7.2) to dilute the presence of M9. Homogenates were resuspended in 300 µl homogenization buffer containing saponin at 1 mg/ml and were homogenized with a Teflon pestle (Kontes glass) for 3 min at 250 rpm at 4 ^{o}C. The homogenate was centrifuged at 650 *g* for 3 min to remove intact worms and nuclear fractions. The supernatant was removed and then centrifuged at 15,000 *g* for 3 min to pellet the mitochondria. The supernatant was removed and discarded, and then the pellet was resuspended in 300 µl homogenization buffer without

ATP (100 mM KCl, 50 mM KH_2PO_4, 50 mM Tris, 5 mM $MgCl_2$, and 1 mM EDTA at pH 7.2). The mitochondrial suspension was further centrifuged at 15,000 *g* for 3 min to wash the mitochondria. The mitochondrial pellet reformed, and after removing the supernatant, the pellet was added to 300 µl of resuspension buffer [human serum albumin, 0.5 mg/ml, 240 mM sucrose, 15 mM KH_2PO_4, 2 mM $Mg(CH_3COO)_2 \times 4\ H_2O$, and 0.5 mM EDTA at pH 7.2] and centrifuged at 15,000 *g* for 3 min. The final mitochondrial pellet was added to 100 µl of resuspension buffer and stored on ice until analysis. To measure CS, 15 µl mitochondrial suspension was added to 185 µl homogenization buffer (50 mM KH2PO4, 1 mM EDTA, and 0.05% Triton X-100) and was homogenized using a glass pestle at 200 rpm for 2 min. The homogenate was then centrifuged at 24,000 *g* before the formation of 5,5'-dithiobis-(2-nitrobenzoic acid)-coenzyme A containing a thiol group (DTNB-CoA-SH) in the supernatant was measured spectrophotometrically at 412 nm to determine CS activity.

RNA interference protocols

Lifespan and healthspan experiments for the RNAi screen were performed using The Infinity Screening System (NemaLife Inc, Texas, USA) – a microfluidics-based lifespan machine. Day 0 adult animals were washed from plates with liquid NGM solution (3 g NaCl, 2.5 g peptone, 1 L H_2O), loaded in a 2.5 ml syringe equipped with a microtube and injected into sterilised Infinity Chips (NemaLife Inc, Texas, USA) pillar-based microfluidic devices, as previously described (2). 100 µl of food solution (liquid NGM containing 20 mg/ml bacteria, 0.6 mM IPTG, 50 µg/ml ampicillin, 100nM AP39 + 0.01% DMSO or 0.01 % DMSO) was then added to the Infinity Chips. Throughout lifespan experiments, the Infinity Chips were stored at 20 °C in a humid chamber to prevent dehydration. To remove progeny, the microfluidic devices were washed with sterile liquid NGM every day for 90s in the Infinity Screening System, for the duration of the worms' lifespan. After each wash the devices were filmed for 90s using an Apple iPod. The food and drug solution were replaced daily in each device. The videos recorded were analysed using the NemaLife software (NemaLife Inc, Texas, USA) to obtain daily numbers for live and moving worms. For all experiments, n≈160 per condition, across 2 biological replicates.

***C. elegans* and bacteria preparation for RNAi screen**

Animals were syncronized to L1 larval stage by gravity flotation and grown for 60 hours on NGM agar plates containing 1 mM IPTG, 50 µg/ml ampicillin and either 100nM AP39

+ 0.01% DMSO or 0.01 % DMSO. The plates were seeded with 200 µL of HT115 (DE3) bacteria expressing double stranded RNA against the genes screened. The Ahringer RNAi library (3) was utilized, purchased from Source Bioscience (Cambridge, UK). HT115 (DE3) bacteria containing the empty L4440 plasmid vector was used as controls. For RNAi preparations, single colonies of bacteria were picked and grown with shaking at 180 rpm, for 16 h, at 37 °C in sterile LB containing 50 µg/ml ampicillin. To induce dsRNA expression, 0.4 mM Isopropyl β-D-1-thiogalactopyranoside (IPTG) was added and the culture was incubated for an additional 2 h at 37 °C with shaking. The culture was then transferred into fresh pre-weighed Falcon tubes and spun down for 15 min at 3000 rpm. The bacterial pellet was weighed and resuspended in liquid NGM to a final concentration of 20 mg/ml bacteria.

NMG plate-based RNAi

Bacterial colonies were grown as detailed above from the MRC Ahringer Library (*elt-6: F52C12.5, elt-3: K02B9.4, bar-1: C54D1.6, ctl-2: Y54G11A.5*). For plate-based RNAi experiments, colonies were picked into LB broth supplemented with 50 µg/mL of ampicillin and grown for 6-8 hours at 37°C with shaking. 200 µL of bacteria was then placed onto NGM plates supplemented with 1 mM IPTG and 50 µg/mL of ampicillin and left to grow for two days at room temperature to allow bacterial IPTG induction. Empty vector (L4440) controls were prepared in the same manner as double-stranded targets for all experiments. RNAi positive controls (*unc-112: C47E8.7*) were performed alongside each experiment to assure gene silencing efficacy.

RNA isolation for next generation sequencing

Synchronised worms were grown on NGM agar plates until young adulthood and treated with AP39 mtH_2S from either L1 or day 0, as described above. On sample collection day, 100 worms were manually picked and added to 1 ml of TRIzol™ Reagent (Thermofisher Scientific, Loughborough, UK). Worms were lysed by pipetting up and down and were incubated at room temperature, for 5 minutes. 0.2 ml of ice cold UltraPure™ Phenol:Chloroform:Isoamyl Alcohol (25:24:1, v/v) (Thermofisher Scientific, Loughborough, UK) was then added and the samples were incubated at room temperature for 3 minutes. The samples were then centrifuged at 4 °C for 15 min, at 12,000 g. The upper aqueous phase containing the RNA was transferred to a fresh tube and an equal volume of 70 % ethanol was added. The samples were mixed by inverting to disperse any

precipitate. The PureLink™ RNA Mini Kit (Thermofisher Scientific, Loughborough, UK) was then used, according to manufacturer instructions, to isolate the RNA. The samples were treated with DNAse I (Thermofisher Scientific, Loughborough, UK) to remove any DNA contamination. The concentration of the isolated RNA was measured using the Nanodrop spectrophotometer (Thermofisher Scientific, Loughborough, UK). N=200 per condition, across two biological replicates.

RNA-seq data generation and pre-processing

Library preparation and sequencing of extracted RNA samples were undertaken by the Beijing Genomics Institute (Hong Kong). All samples had good RNA quality (RNA Integrity Number ≥ 7 and 28S/18S ≥ 1, as determined using the Agilent 2100 Bioanalyzer system; Agilent Technologies) and were sequenced to generate strand-specific (second strand cDNA synthesis with dUTP) 100 bp paired-end reads using the DNBSEQ platform. Reads were filtered to remove adaptor sequences, contamination and low-quality reads, with all cleaned reads deemed to be of good quality (no over-represented sequences or adapter sequences and median per base quality scores always > 30, as determined using FastQC; Babraham Bioinformatics). Clean reads were subsequently pseudo-aligned to the *C. elegans* reference transcriptome (Ensembl release 106, Wbcel235 cDNA FASTA file) using Kallisto (v0.46.1)(4) to estimate transcript-level abundances, from which gene-level counts were inferred via the tximport package for R (5). Genes with consistently low expression (count < 10 in every sample) were then removed, leaving 10,451 genes for downstream analyses.

RNA-seq data analyses

The DESeq2 package for R (6) was used to test for differential gene expression, with comparisons made between day 0 wild-type worms and wild-type/treated worms from each of the other two time points, as well as directly between conditions at each of the three time points. In each case, the 'ashr' adaptive shrinkage method was applied to log2 fold-change estimates (7) and the Benjamini-Hochberg procedure used to control for false discovery rate (FDR), with genes defined as significantly differentially expressed when FDR < 0.05. To further elucidate time-specific condition effect patterns, the 'degPatterns' algorithm of the DEGreport R package was applied to normalized counts (obtained *via* variance stabilizing transformation) to group by expression profile any gene differentially regulated between treatments at day 0 and/or: differentially expressed in at least one

treatment at one or more time point *vs*. day 0 wild-types *and* differentially expressed directly between at least one treatment pair at one or more time points. During the clustering process, genes outlier of cluster distribution were removed, and a default minimum cluster size of 15 genes applied. Functional enrichment analysis of defined gene clusters was consequently undertaken using the gprofiler2 package for R (8), with the following data sources utilized: each Gene Ontology (GO) category (biological process, BP; cellular component, CC; molecular function, MF), the Kyoto Encyclopedia of Gene and Genomes (KEGG) pathway database, and regulatory motif matches from TRANSFAC. In each case, all genes that were tested for differential expression were input as background and the annotated statistical domain scope option employed, with enriched terms selected as those with an FDR < 0.05 enriched for at least 2 genes. Each gene cluster was also input into the Online Search Tool for Retrieval of Interacting Genes/Proteins (STRING, v11.5; (9)) to infer respective protein-protein interaction (PPI) networks, with default confidence score of > 0.4 used to define connections, as quantified using all active interaction sources *excluding* text-mining. Network components were ranked by node degree to deduce the most highly connected ('hub') components within each network.

7-azido-4-methylcoumarin measures of total sulfide levels

Sulfide content was assessed as described previously (10, 11). 120 animals were picked into M9 buffer in 1.5 mL low-bind eppendorphs and washed three times with 1 mL of sterile M9 by flotation to clear bacterial debris and progeny. Samples were snap-frozen in a final 40 µL volume of M9 and stored at -80°C until analysis (within 1 week). On the day of analysis, samples were freeze-thawed three times in liquid nitrogen with three 5-minute sonication cycles after each thaw (VWR, UK) to aid cuticle rupture. Samples were then centrifuged at 14,000 g for 15 minutes at 4 °C to pellet animal exoskeletons, recovering supernatants and storing on ice for 5 minutes for downstream analysis. Stocks of the H_2S-sensitive fluorogenic probe 7-azido-4-methylcoumarin (AzMC) were prepared in 100 % DMSO at 10 mM, where 20 µL of protein supernatant was incubated with 140 µL of diluted AzMC at a final concentration of 50 µM (in 0.5% DMSO) at 37°C for 1-hour in opaque eppendorphs with shaking at 200 RPM. Samples were then measured in triplicate in black 96-well plates on a BMG FLUOstar plate reader with excitation and emissions of 355/ 460nm. All readings were normalised to protein content that was derived from the same sample lysate assessed by the DC-protein assay as per the manufacturer's instructions.

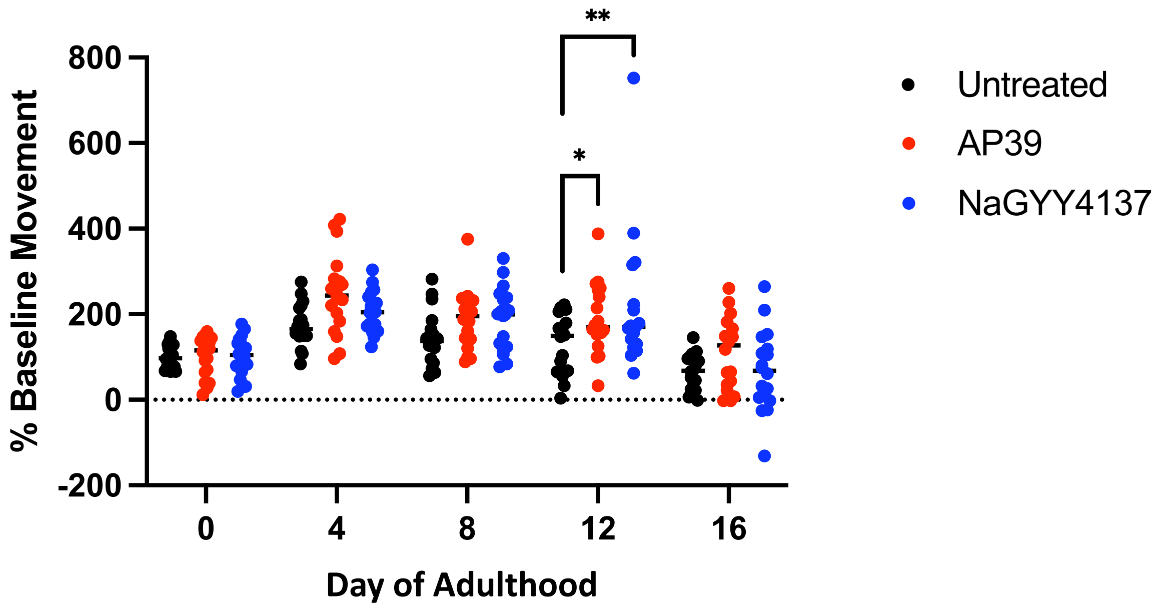

Supplemental Figure 1. Temporal changes in developmental H_2S treated *C. elegans* movement rates. Animal movement rates in response to mitochondria-targeting H_2S (AP39) and un-targeted H_2S (GYY4137) donors. H_2S-induced improved healthspan is accounted for by specific increases at later life (day 12) time points when individual values for % movement increase from baseline, at each time point are analyzed by two-way repeated measures ANOVA. * , P<0.05; **, P<0.01; ****, P<0.0001.

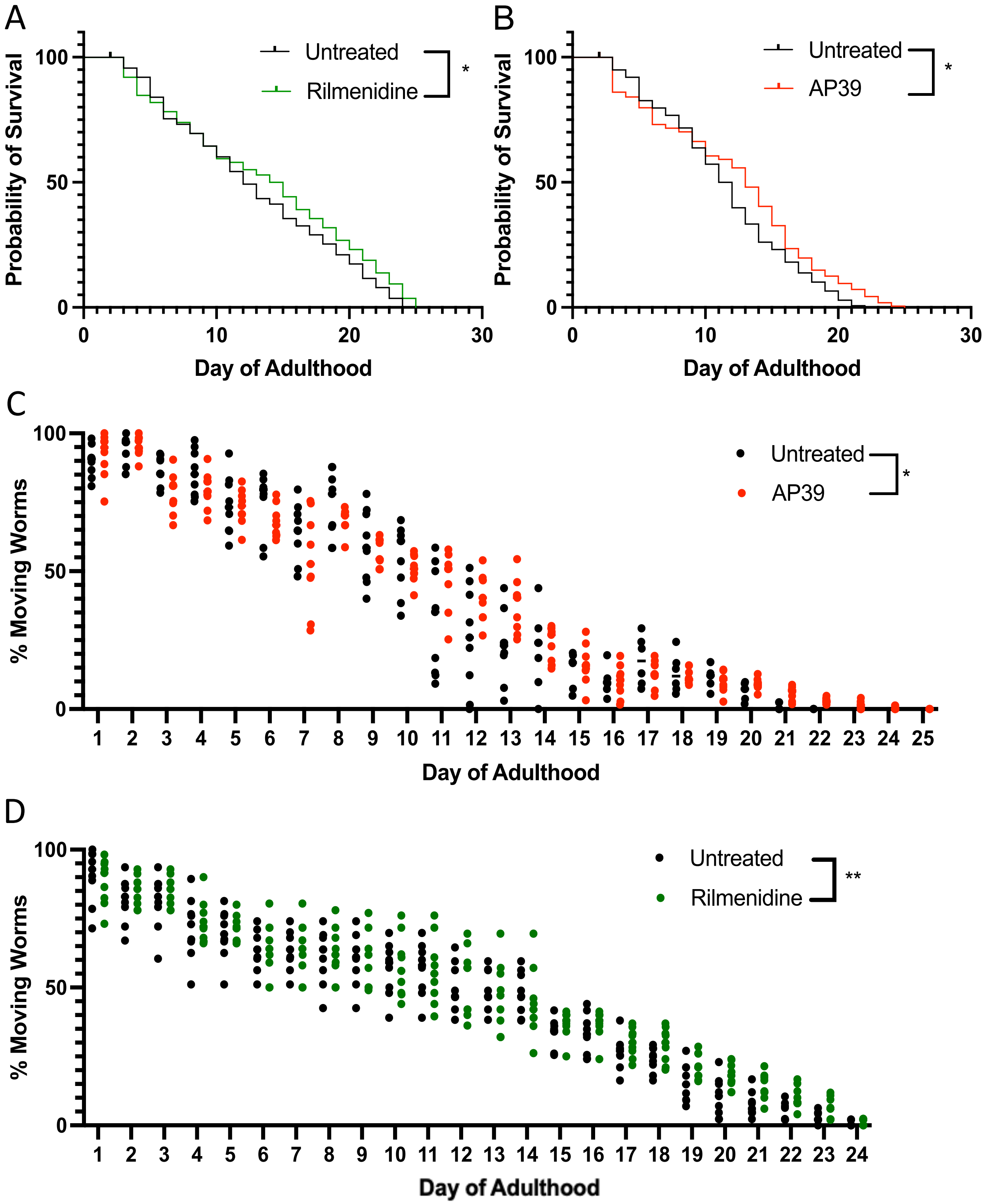

Supplemental Figure 2. AP39 lifespan and healthspan extension is comparable to a published positive control compound. (A) *C. elegans* lifespan is significantly increased after treatment with mitochondria-targeted H_2S donor, AP39 (untreated = 0.01% DMSO, n ≅ 200 animals per treatment, across 2 or 3 biological repeats). **(B)** Likewise, a recently published life-extension drug, rilmenidine, extends lifespan to a similar magnitude as AP39 (untreated = 1% DMSO, n ≅ 200 animals per treatment, across 2 or 3 biological repeats). **(C)** Similarly, healthspan is extended in AP39-treated animals (n ≅ 200 animals per treatment, across 3 technical and 2 or 3 biological repeats) and **(D)** rilmenidine-treated animals (n ≅ 200 animals across 3 technical and 2 or 3 biological repeats). * (P<0.01). ** (P<0.001).

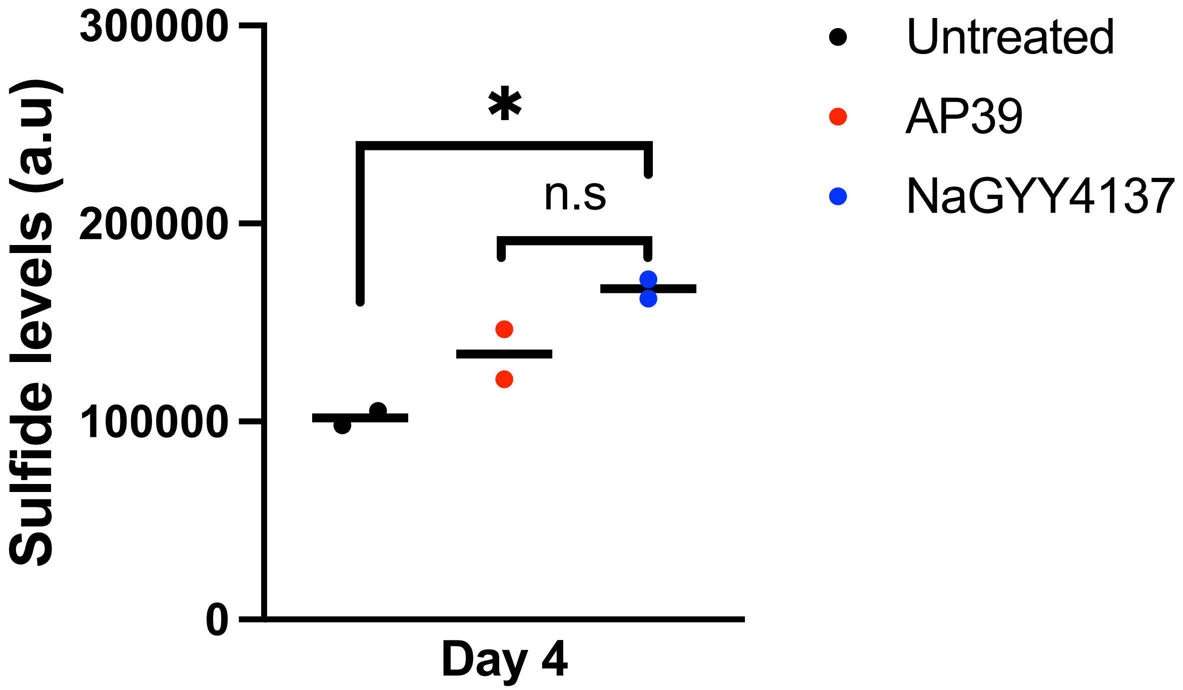

Supplemental Figure 3. Total sulfide levels do not differ between AP39 and NaGYY4137 treatments in *C. elegans.* Sulfide levels between animals treated with AP39 or NaGYY4137 from L1 larval are not different at day 4 post-adulthood (*P*>0.05), despite elevated sulfide levels in NaGYY4137 treated animals. Data represents ~120 animals per biological replicate, across two biological replicates (~240 animals in total). a.u, arbitrary unit; ns, non-significant. *, *P*<0.05.

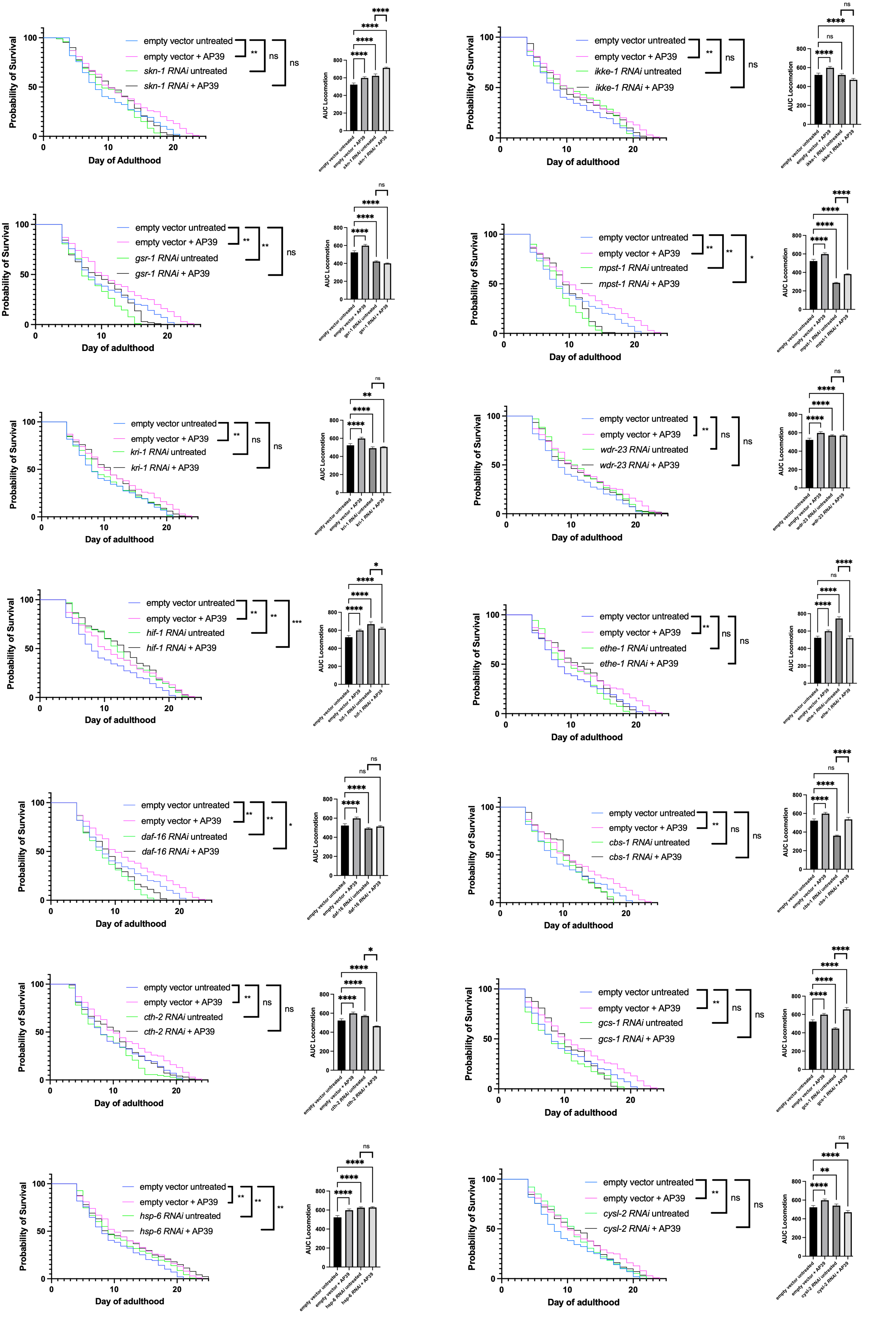
Probability of Survival
Day of Adulthood
empty vector untreated
empty vector + AP39
skn-1 RNAi untreated
skn-1 RNAi + AP39
**
ns
ns
AUC Locomotion
****
****
****
****
Probability of Survival
Day of adulthood
empty vector untreated
empty vector + AP39
ikke-1 RNAi untreated
ikke-1 RNAi + AP39
**
ns
ns
AUC Locomotion
****
ns
****
ns
Probability of Survival
Day of adulthood
empty vector untreated
empty vector + AP39
gsr-1 RNAi untreated
gsr-1 RNAi + AP39
**
**
ns
AUC Locomotion
****
****
****
ns
Probability of Survival
Day of adulthood
empty vector untreated
empty vector + AP39
mpst-1 RNAi untreated
mpst-1 RNAi + AP39
**
**
*
AUC Locomotion
****
****
****
****
Probability of Survival
Day of adulthood
empty vector untreated
empty vector + AP39
kri-1 RNAi untreated
kri-1 RNAi + AP39
**
ns
ns
AUC Locomotion
****
****
**
ns
Probability of Survival
Day of adulthood
empty vector untreated
empty vector + AP39
wdr-23 RNAi untreated
wdr-23 RNAi + AP39
**
ns
ns
AUC Locomotion
****
****
****
ns
Probability of Survival
Day of adulthood
empty vector untreated
empty vector + AP39
hif-1 RNAi untreated
hif-1 RNAi + AP39
**
**
***
AUC Locomotion
****
****
****
*
Probability of Survival
Day of adulthood
empty vector untreated
empty vector + AP39
ethe-1 RNAi untreated
ethe-1 RNAi + AP39
**
ns
ns
AUC Locomotion
****
****
ns
****
Probability of Survival
Day of adulthood
empty vector untreated
empty vector + AP39
daf-16 RNAi untreated
daf-16 RNAi + AP39
**
**
*
AUC Locomotion
****
****
ns
ns
Probability of Survival
Day of adulthood
empty vector untreated
empty vector + AP39
cbs-1 RNAi untreated
cbs-1 RNAi + AP39
**
ns
ns
AUC Locomotion
****
****
ns
****
Probability of Survival
Day of adulthood
empty vector untreated
empty vector + AP39
cth-2 RNAi untreated
cth-2 RNAi + AP39
**
ns
ns
AUC Locomotion
****
****
****
*
Probability of Survival
Day of adulthood
empty vector untreated
empty vector + AP39
gcs-1 RNAi untreated
gcs-1 RNAi + AP39
**
ns
ns
AUC Locomotion
****
****
****
****
Probability of Survival
Day of adulthood
empty vector untreated
empty vector + AP39
hsp-6 RNAi untreated
hsp-6 RNAi + AP39
**
**
**
AUC Locomotion
****
****
****
ns
Probability of Survival
Day of adulthood
empty vector untreated
empty vector + AP39
cysl-2 RNAi untreated
cysl-2 RNAi + AP39
**
ns
ns
AUC Locomotion
****
**
****
ns

Supplemental Figure 4. Lifespan and healthspan responses to combined mitochondria-targeted H_2S and targeted gene knockdowns. Full lifespan curves and healthspan responses, represented as area under the curve (AUC) of percentage movement rate changes from day 0 post-adulthood baseline (movement rates measured at days 0, 2, 4, 8 and 12 post-adulthood). Data represents ~160 animals across biological duplicates per condition. **, $P<0.01$; ****, $P<0.0001$.

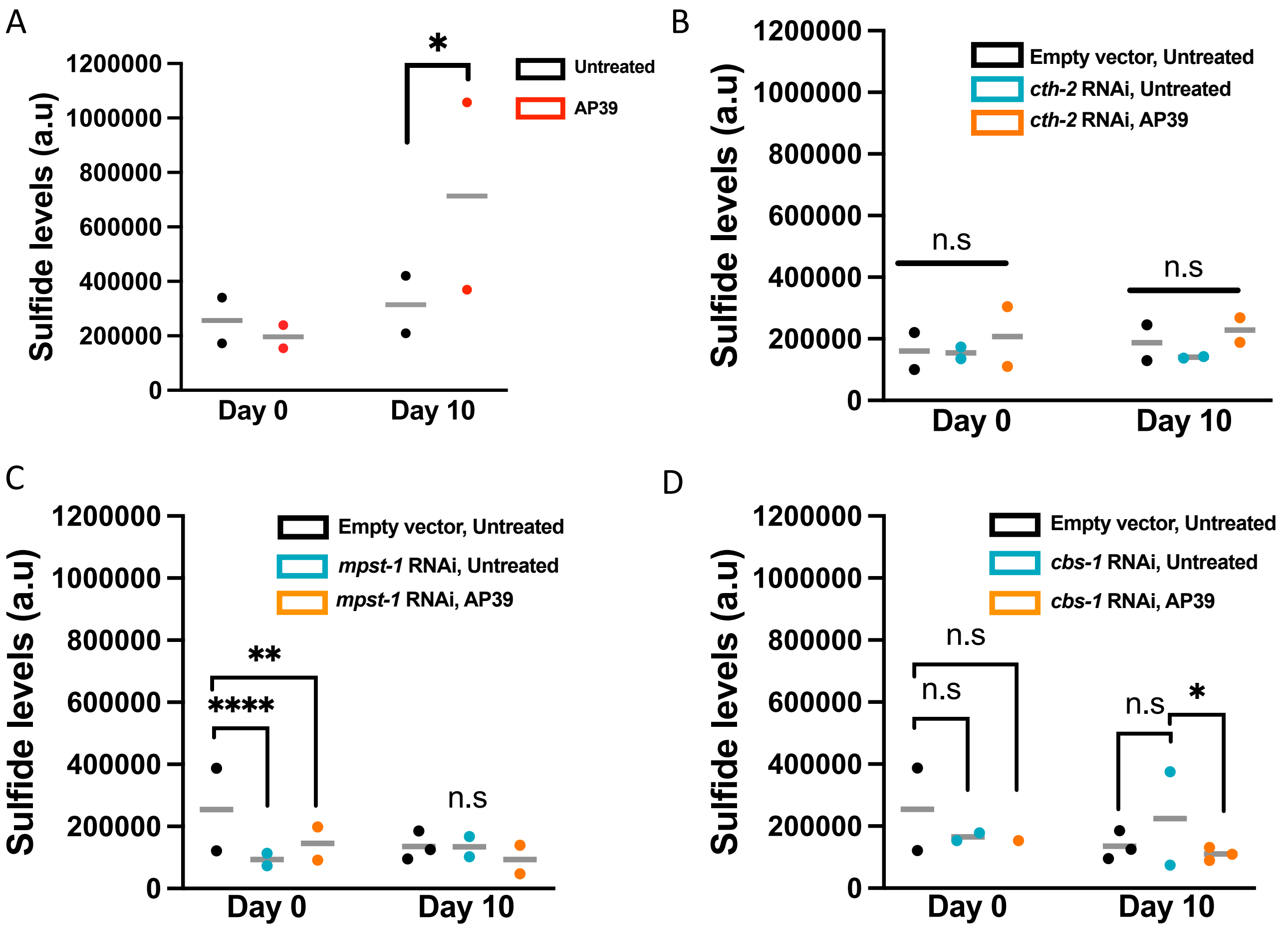

Supplemental Figure 5. mtH_2S increases total sulfide levels in older *C. elegans* and requires endogenous H_2S producing enzymes. (A) H_2S levels at day 0 and 10 post-adulthood in untreated and AP39 treated animals from the L1 larval stage, where AP39 increases later-life sulfide levels. **(B)** Sulfide levels at days 0 and 10 post-adulthood during RNAi against *cth-2*, **(C)** *mpst-1* or **(D)** *cbs-1* with RNAi alone or combined RNAi + AP39 treatment from L1. Data represents ~120 animals across 3 technical repeats, for each of 2 biological repeats. a.u, arbitrary unit; ns, non-significant. * denotes significant effect of treatment for within-day comparisons against untreated animals (*, P<0.05; **, P<0.01; ****, P<0.0001).

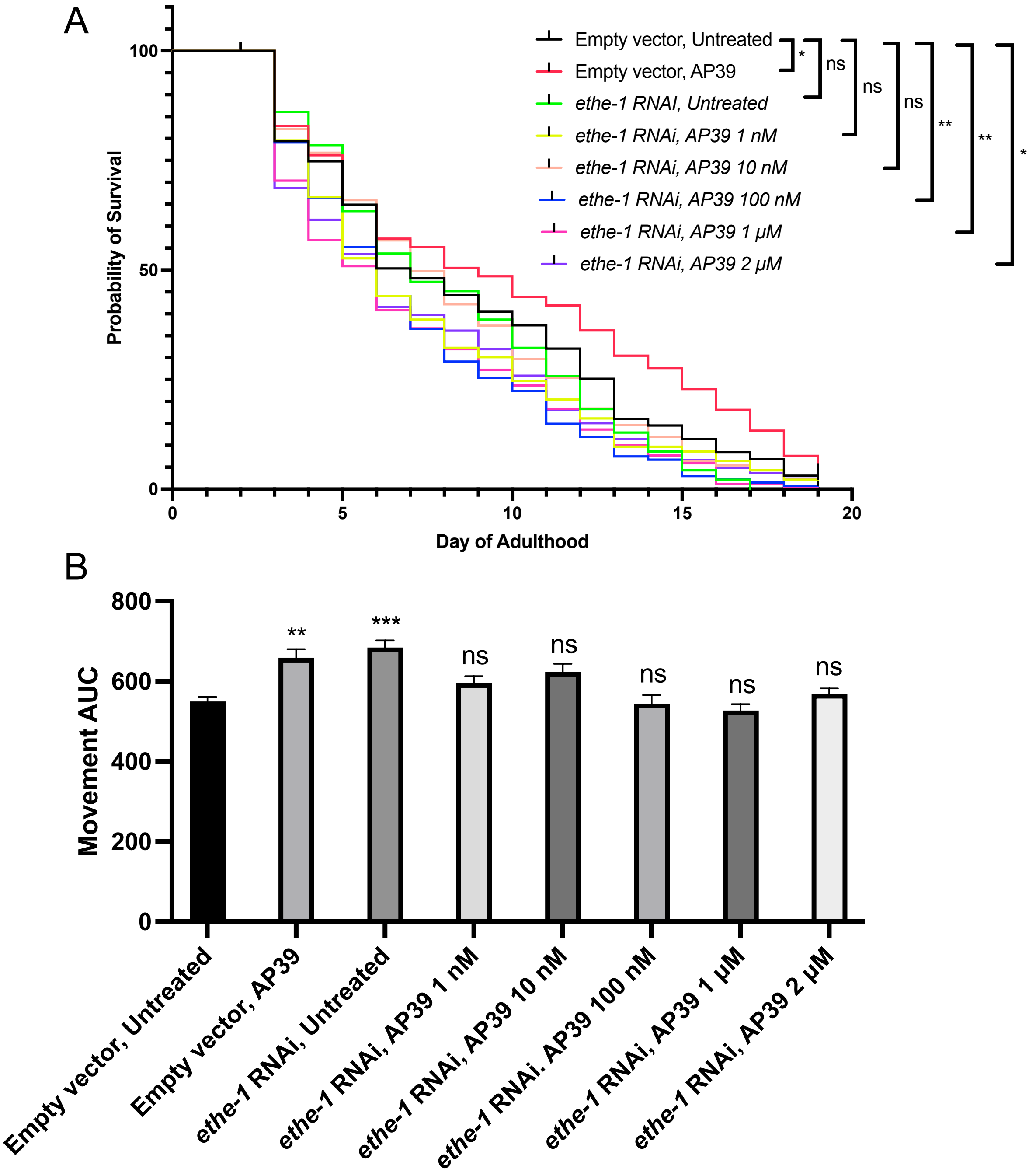

Supplemental Figure 6. Toxic lifespan effects of combined AP39 plus *ethe-1* knockdown are dose-dependent. (A) *ethe-1 RNAi* + AP39 treatment shortens lifespan at 100 nM, 1 μM, and 2 μM, but not at 1 nM or 10 nM of AP39 (~200 animals per treatment, across 2 or 3 biological repeats). **(B)** Animal movement rate is increased with AP39 treatment and *ethe-1* RNAi. However, AP39-treatment in *ethe-1* RNAi worms suppresses the increased movement rate at all concentrations tested between 1 nM and 2 μM (~200 animals per treatment, across 3 technical and 2 or 3 biological repeats). * (P<0.01). ** (P<0.001), *** (P<0.0001).

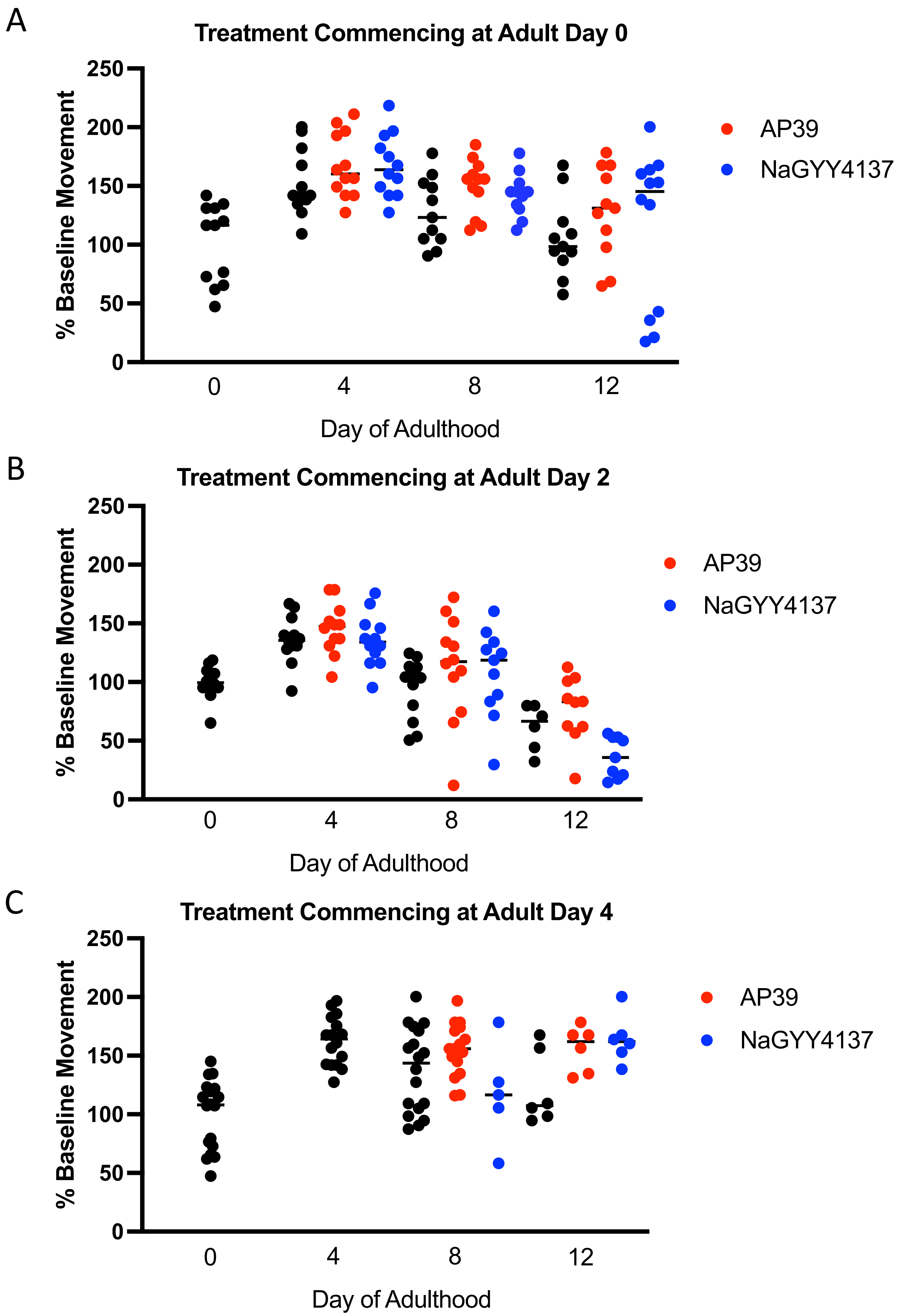

Supplemental Figure 7. Temporal changes in adult H_2S treated *C. elegans* movement rates. Animal movement rates in response to mitochondria-targeting H_2S (AP39) and un-targeted H_2S (GYY4137) donors. Statistics represent area under the curve analysis of individual % changes in movement from baseline values at each time point. * , *P*<0.05; **, *P*<0.01; ***, *P*<0.001; ****, *P*<0.0001.

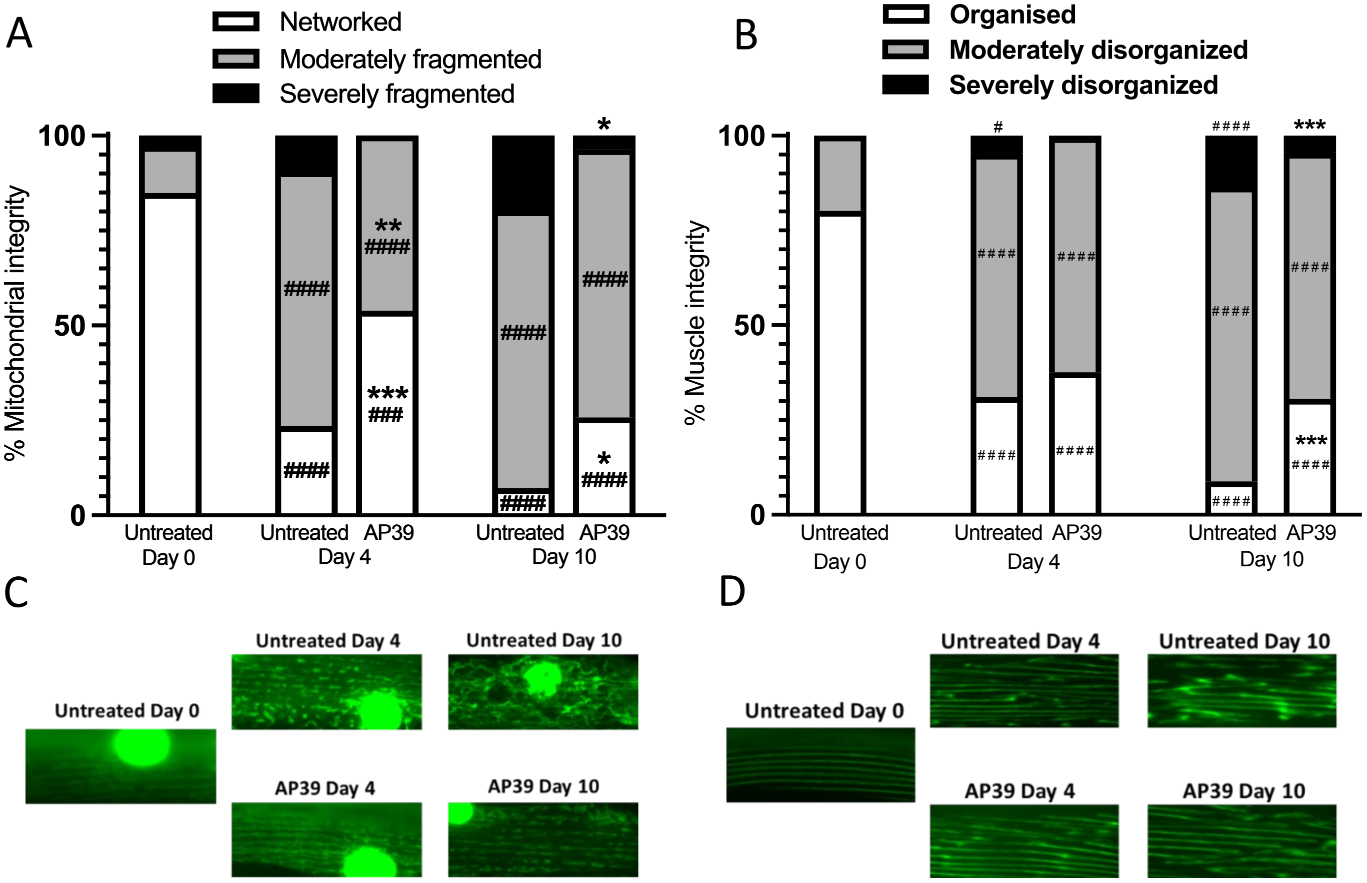

Supplemental Figure 8. Adult onset mtH_2S improves age-related deterioration of mitochondrial and myofibrillar networks. (A) Age-related deterioration of well-networked mitochondria present by day 4 post-adulthood in both untreated and AP39 treated conditions compared to day 0 adults, which is attenuated by AP39 at both day 4 and day 10 post-adulthood. Adult-onset AP39 treatment also reduces the prevalence of severe mitochondrial fragmentation in day 10 animals. **(B)** Representative images of mitochondrial structure. **(C)** A significant loss of myofibrillar organization is present in both untreated and AP39 treated animals at days 4 and 10 post-adulthood compared to day 0 adults. AP39 preserves the proportion of organized sarcomeres at day 10 compared to untreated animals and reduces the proportion of severely disorganized sarcomeres. **(D)** Representative images of myofibrillar organization. Data represent 40-60 animals from two biological replicates for both mitochondrial and myofibrillar data, from ~300 muscle cells per condition, per time point. # denotes significance of aging effect compared to day 0 animals (#, *P*<0.05; ####, *P*<0.0001). * denotes significant effect of treatment for within-day comparisons against untreated animals (*, *P*<0.05; **, *P*<0.01; ***, *P*<0.001).

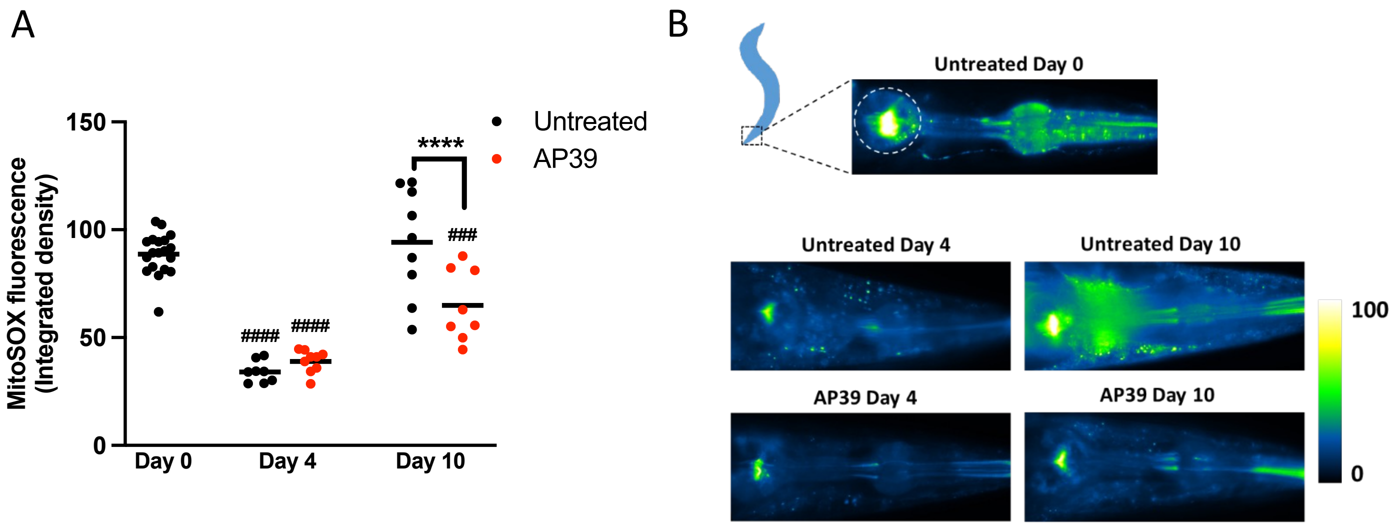

Supplemental Figure 9. Age-related increases in mitochondrial superoxide levels are lowered with adult-onset mtH_2S treatment. (A) Fluorescent superoxide production within the pharyngeal terminal bulb decreased from young adult stage worms to 4 days post-adulthood in both untreated and AP39 treatments. Superoxide production subsequently increased in day 10 older animals, which was repressed with adult onset AP39 treatment. **(B)** Representative images of pharyngeal terminal bulb fluorescence. White dashed circle signifies the area of fluorescence quantification. Data represent two biological repeats where images were taken from ~15-30 animals per condition, per time point. # denote age-related declines to day 0 untreated animals (###, *P*<0.001; ####, *P*<0.0001). * denotes significant effect of treatment for within-day comparisons against untreated animals (****, *P*<0.0001).

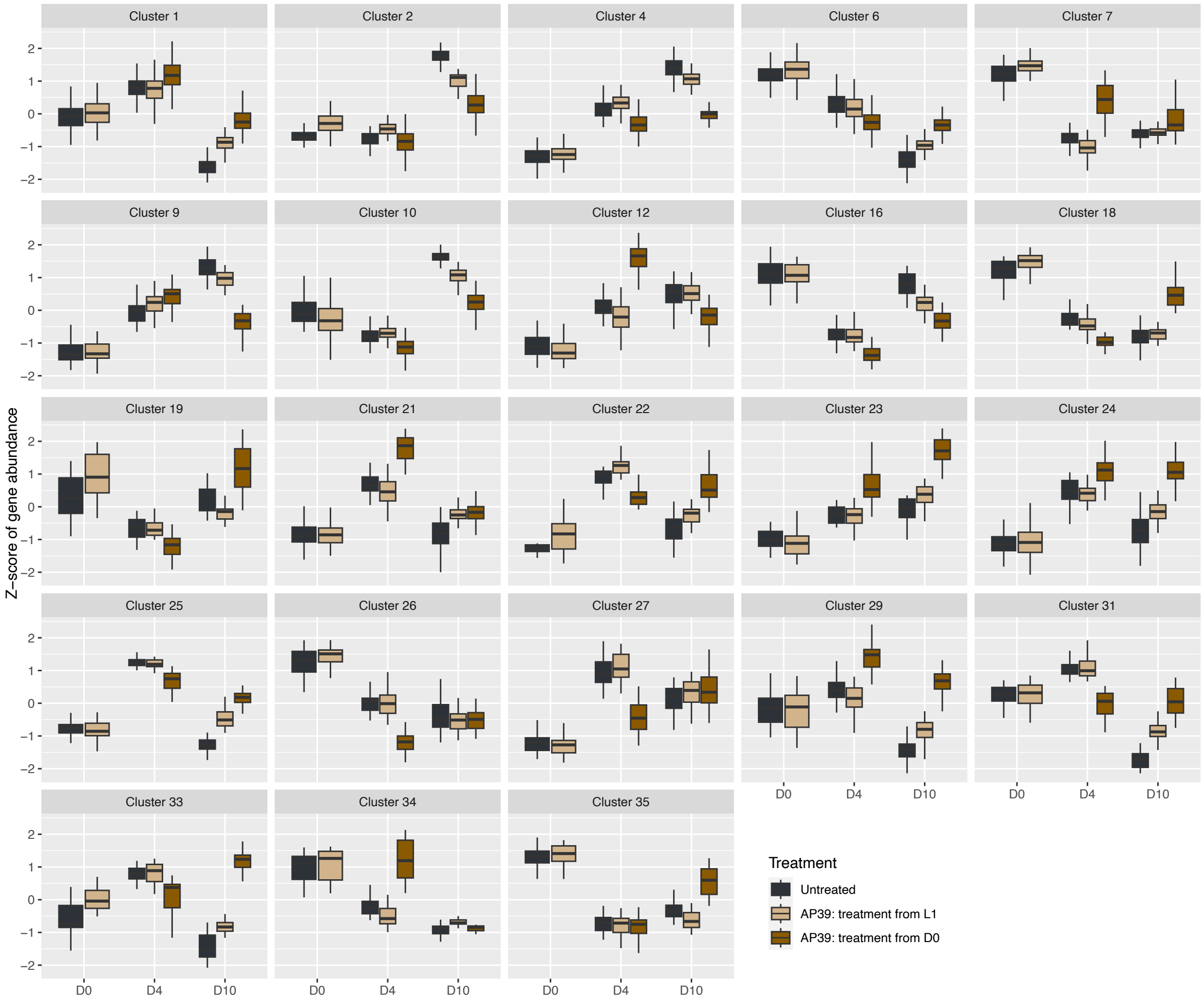

Supplemental Figure 10. Boxplots depicting time/condition expression trends Expression trends are represented as Z-score of gene abundance for clusters of differentially expressed genes < 200 genes in size (i.e., clusters not displayed in main Figure X). WT = wild-type. D0, D4 and D10 = days 0, 4 and 10 post-adulthood, respectively.

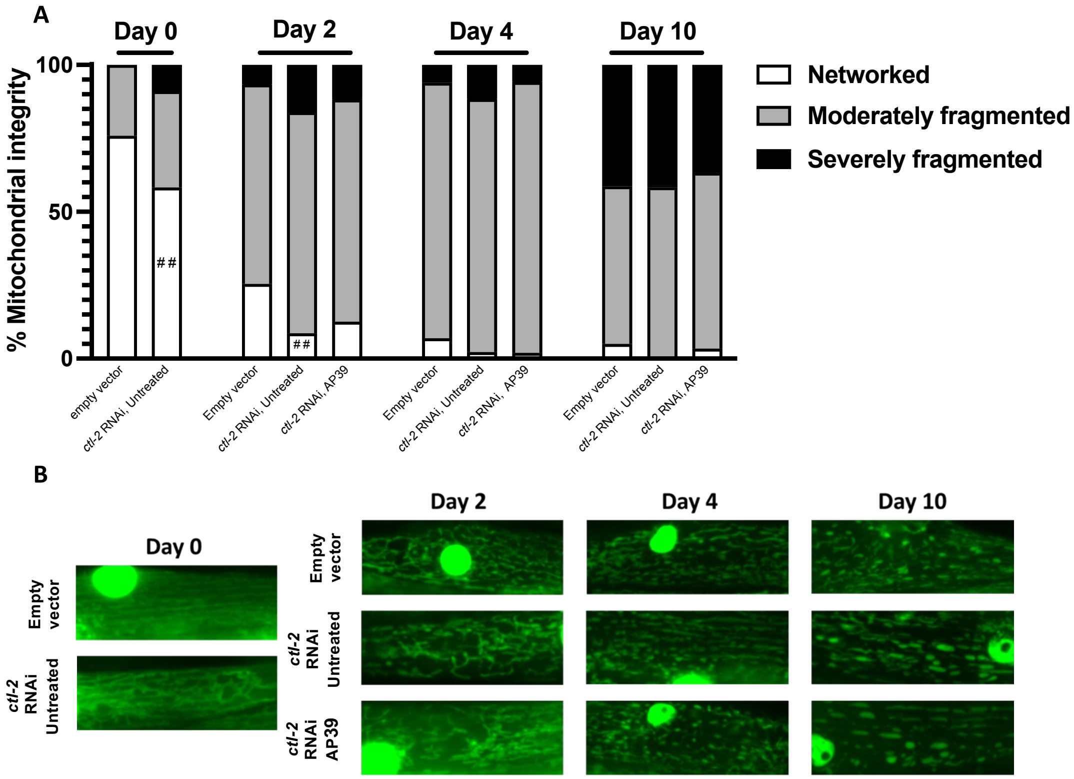

Supplemental Figure 11. A peroxisome-related hub gene is required for the mitochondria-preserving effects of adult mtH_2S treatment. (A) RNAi knockdown of the peroxisomal catalase *ctl-2* exacerbates mitochondrial fragmentation in early life and prevents the mitochondria-preserving effects of adult onset AP39 at day 4 and 10 post-adulthood. **(B)** Representative images of mitochondrial localization. Data represent 40-60 animals spanning ~300 muscle cells from two biological repeats. # denotes significant effect of aging from day 0 empty vector animals (##, P<0.01).

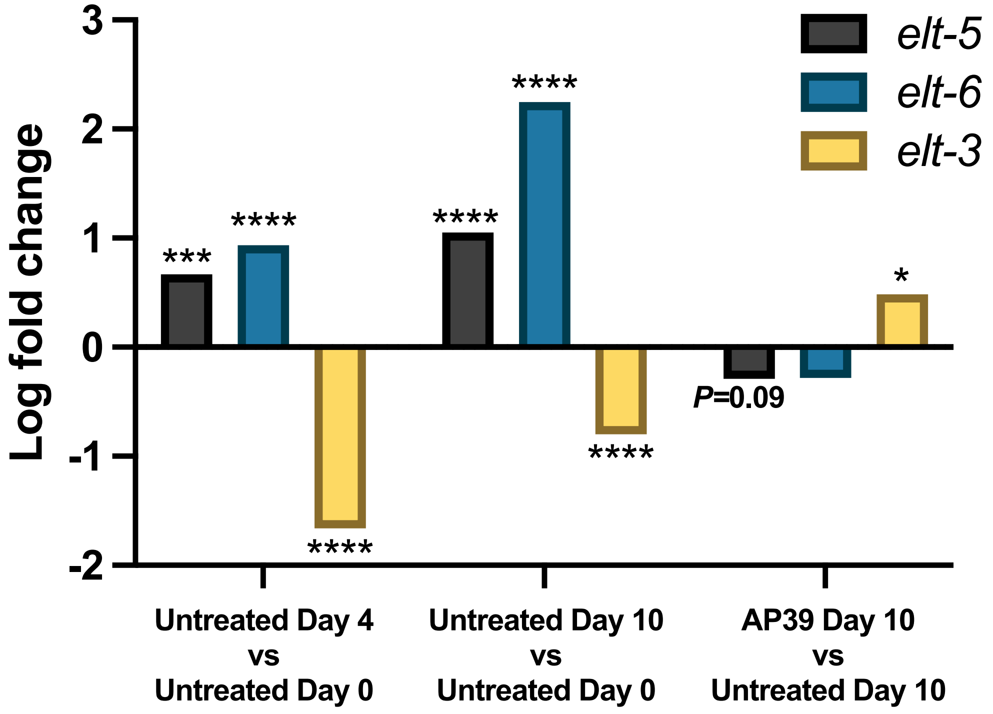

Supplemental Figure 12. Effects of aging and adult onset mtH_2S on gene expression of the ELT-5, 6 and 3 GATA transcription factor circuit. Transcriptomic RNA sequencing analysis show increased *elt-5* and *elt*-6, and reduced *elt-3* gene expression at day 4 and 10 post-adulthood in untreated *C. elegans*. Adult onset AP39 treatment reverse this pattern at day 10 post-adulthood. * denotes significant difference from untreated day 0 (untreated data) or day 10 (AP39 data) animals. *, *P*<0.05; ***, *P*<0.001; ****, *P*<0.0001.

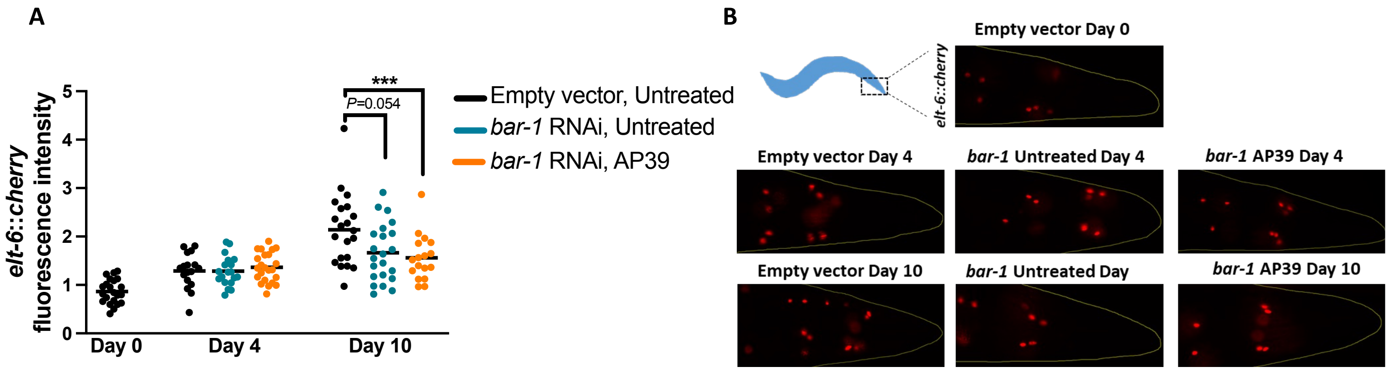

Supplemental Figure 13. The cytoskeletal and mitochondria-localized BAR-1 independently modulates ELT-6 expression. (A) The age-related increase in ELT-6 expression is reduced with *bar-1* RNAi, which is not synergistically lowered with combined *bar-1* RNAi + adult onset AP39 treatment. **(B)** Representative ELT-6 fluorescent images. Data represent 40-60 animals per condition, per time point across two biological repeats. * denotes significant effect of treatment for within-day comparisons against untreated animals (***, P<0.001).

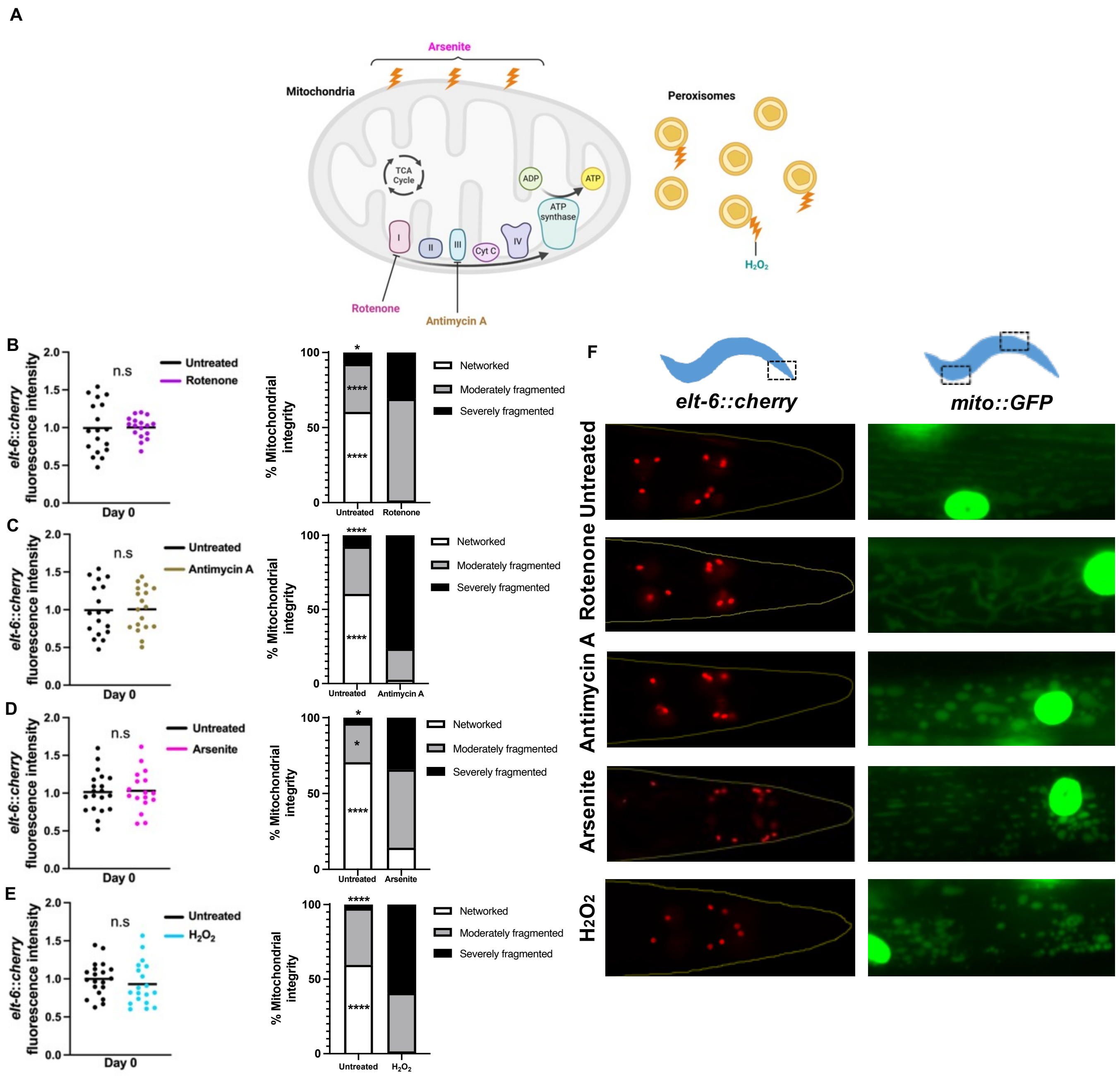

Supplemental Figure 14. Mitochondrial toxins do not influence ELT-6 expression despite severe mitochondrial fragmentation. (A) Schematic detailing sites of action of inhibitor-induced mitochondrial dysfunction and peroxisomal stress employed in *elt-6::mCherry* animals at day 0 of adulthood. **(B – E)** Effects of acute treatment with rotenone (50μM), antimycin A (150μM), arsenite (5mM) or hydrogen peroxide (H2O2, 100μM) on ELT-6 expression (left column) and mitochondrial integrity (right column). (F) Representative images for *elt-6::mCherry* (left column) and body-wall muscle mitochondria (right column). Data represent 20-25 animals per condition spanning ~150 muscle cells. * denote significance between mitochondrial structural classes of untreated and inhibitor treated animals (*, $P<0.05$; ****, $P<0.0001$).

Supplemental Table 1. Raw RNA-seq results and outputs.

References

1. T. Etheridge, *et al.*, The integrin-adhesome is required to maintain muscle structure, mitochondrial ATP production, and movement forces in Caenorhabditis elegans. *Faseb J* 29, 1235–1246 (2015).

2. M. Rahman, *et al.*, NemaLife chip: a micropillar-based microfluidic culture device optimized for aging studies in crawling C. elegans. *Sci Rep-uk* 10, 16190 (2020).

3. A. G. Fraser, *et al.*, Functional genomic analysis of C. elegans chromosome I by systematic RNA interference. *Nature* 408, 325–330 (2000).

4. N. L. Bray, H. Pimentel, P. Melsted, L. Pachter, Near-optimal probabilistic RNA-seq quantification. *Nat Biotechnol* 34, 525–527 (2016).

5. C. Soneson, M. I. Love, M. D. Robinson, Differential analyses for RNA-seq: transcript-level estimates improve gene-level inferences. *F1000research* 4, 1521 (2016).

6. M. I. Love, W. Huber, S. Anders, Moderated estimation of fold change and dispersion for RNA-seq data with DESeq2. *Genome Biol* 15, 550 (2014).

7. M. Stephens, False discovery rates: a new deal. *Biostatistics* 18, kxw041 (2016).

8. L. Kolberg, U. Raudvere, I. Kuzmin, J. Vilo, H. Peterson, gprofiler2 -- an R package for gene list functional enrichment analysis and namespace conversion toolset g:Profiler. *F1000research* 9, ELIXIR-709 (2020).

9. D. Szklarczyk, *et al.*, STRING v11: protein–protein association networks with increased coverage, supporting functional discovery in genome-wide experimental datasets. *Nucleic Acids Res* 47, gky1131 (2018).

10. R. A. Ellwood, *et al.*, Mitochondrial hydrogen sulfide supplementation improves health in the C. elegans Duchenne muscular dystrophy model. *Proc National Acad Sci* 118, e2018342118 (2021).

11. R. A. Ellwood, *et al.*, Sulfur amino acid supplementation displays therapeutic potential in a C. elegans model of Duchenne muscular dystrophy. *Commun Biology* 5, 1255 (2022).
